# Key role of quinone in the mechanism of respiratory complex I

**DOI:** 10.1038/s41467-020-17957-0

**Published:** 2020-08-18

**Authors:** Javier Gutiérrez-Fernández, Karol Kaszuba, Gurdeep S. Minhas, Rozbeh Baradaran, Margherita Tambalo, David T. Gallagher, Leonid A. Sazanov

**Affiliations:** 1 Institute of Science and Technology Austria, Am Campus 1, A-3400 Klosterneuburg, Austria; 2 Medical Research Council Mitochondrial Biology Unit, Keith Peters Building, Hills rd, Cambridge, CB2 0XY UK; 3 Present Address: Sosei Heptares, Steinmetz Building, Granta Park, Cambridge, CB21 6DG UK; 4 Present Address: Science for Life Laboratory, Department of Biochemistry and Biophysics, Stockholm University, 17165 Solna, Sweden

**Keywords:** Enzyme mechanisms, Cryoelectron microscopy, X-ray crystallography, Bioenergetics

## Abstract

Complex I is the first and the largest enzyme of respiratory chains in bacteria and mitochondria. The mechanism which couples spatially separated transfer of electrons to proton translocation in complex I is not known. Here we report five crystal structures of *T. thermophilus* enzyme in complex with NADH or quinone-like compounds. We also determined cryo-EM structures of major and minor native states of the complex, differing in the position of the peripheral arm. Crystal structures show that binding of quinone-like compounds (but not of NADH) leads to a related global conformational change, accompanied by local re-arrangements propagating from the quinone site to the nearest proton channel. Normal mode and molecular dynamics analyses indicate that these are likely to represent the first steps in the proton translocation mechanism. Our results suggest that quinone binding and chemistry play a key role in the coupling mechanism of complex I.

## Abstract (web summary)

Complex I (NADH:ubiquinone oxidoreductase) is the first enzyme of the respiratory chain in bacteria and mitochondria. Here, the authors present cryo-EM and crystal structures of *T. thermophilus* complex I in different conformational states and further analyse them by Normal Mode Analysis and molecular dynamics simulations and conclude that quinone redox reactions are important for the coupling mechanism of complex I.

## Introduction

Complex I (NADH:ubiquinone (UQ) oxidoreductase) is the first and the largest enzyme of the respiratory chain, catalysing the transfer of two electrons from NADH to UQ, coupled to the translocation of four protons across the bacterial or inner mitochondrial membrane^1–5^. Recent studies on respirasomes suggest that complex I forms supercomplexes with other elements of the respiratory chain, able to perform the electron transport in a more packed and controlled environment^6–8^. In addition, there is a plethora of diseases related to complex I malfunctioning and/or caused by numerous amino acid mutations found in nearly all of 45 subunits of this enzyme^9–11^. Bacterial enzyme represents a “minimal” version of complex I with 14 conserved “core” subunits of about 0.5 MDa in total^5^. More elaborate mitochondrial enzyme contains about 30 additional “supernumerary” subunits bringing the total molecular weight of the complex to about 1 MDa. We obtained initial structural information about complex I by X-ray crystallography of separate bacterial complex I domains, followed by the structure of the entire *Thermus thermophilus* complex^12–16^. The first complete structure of the mammalian (ovine) enzyme was solved by cryo-electron microscopy (cryo-EM)^17^. Further mitochondrial mammalian and yeast *Yarrowia lipolytica* complex I crystal and cryo-EM structures were determined in recent years^7,18–21^. *T. thermophilus* complex I crystal structure, comprising 16 subunits of a total molecular weight of 550 kDa, is the most complete high-resolution structure^5,16^ and it provides a reliable basis and a simpler model to study the catalytic mechanism as well as the effect of substrates and inhibitors on this enzyme.

**Fig. 1:**
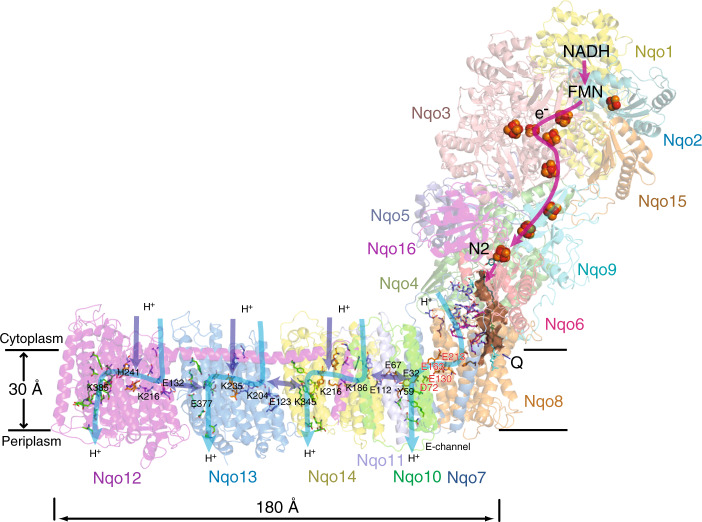
Overall structure and organization of *Thermus thermophilus* complex I. The electron transfer pathway in the peripheral arm (PA) and putative proton translocation pathways in the membrane domain (MD) are indicated by arrows. Complex I subunits are coloured individually and labelled. Fe–S clusters are represented as spheres, with cluster N2 labelled. The quinone is shown as cyan sticks within the quinone-binding cavity (brown surface). The position of its headgroup is based on our DQ data, but the entire ubiquinone tail was modelled to illustrate the extent of the native molecule. In the proton translocation channels, polar residues lining the channels are shown as sticks with carbon in dark blue for the first (N-terminal) half-channel, in green for the second (C-terminal) half-channel, and in orange for the connecting residues. Key residues for antiporter-like subunits, GluTM5 and LysTM7 from the first half-channel, Lys/HisTM8 from the connection, and Lys/GluTM12 from the second half-channel, are labelled. Residues playing similar roles in the E-channel are also labelled (Glu/Asp quartet in red). The central hydrophilic axis extends from the Q site to the tip of the MD along these indicated key residues. The residues connecting quinone cavity to the E-channel are in magenta. Proton translocation pathways through symmetry-related half-channels are indicated by blue arrows, and additional possible paths (extra entry sites and inter-subunit transfer) by violet arrows.

In bacterial complex I the 14 “core” subunits, necessary and sufficient for complex I function, are shared equally between the peripheral and membrane arms, together forming an L-shaped molecule. The *T. thermophilus* peripheral arm (PA) contains nine subunits: core Nqo1–6, Nqo9 (*Thermus* nomenclature) and two additional *Thermus*-specific subunits (Nqo15/16). All known co-factors of complex I reside in the PA: the primary electron acceptor flavin-mononucleotide (FMN) is connected by the redox chain of seven Fe–S clusters to the quinone-binding site (Q-site) at the interface with the membrane domain (Fig. 1). Subunits Nqo1–3 form the NADH-oxidizing domain, while subunits Nqo4–6 form the connecting domain, providing a link to the membrane domain (MD), which comprises seven subunits: Nqo7–8 and Nqo10–14, with a total of 64 transmembrane helices (TM). Antiporter-like subunits Nqo12, Nqo13, and Nqo14 are homologous to each other and to the bacterial cation/H^+^ Mrp antiporter complex subunits MrpA/D, containing 14 conserved TMs each. In these subunits, two sets of five helices (TMs 4–8 and TMs 9–13) are related to each other by internal symmetry and contain key conserved charged residues (Lys/Glu) sitting mid-membrane on a break in symmetry-related TMs 7 and 12. Each set likely comprises half of a proton channel, with the N-terminal set linked to the bacterial cytoplasm/mitochondrial matrix and the C-terminal set linked to the periplasm/inter-membrane space (IMS). Two half-channels are linked in the middle of the membrane into a single trans-membrane channel by additional conserved charged residues, one of them sitting on the break in TM8. Thus, key residues participating in proton translocation mostly sit mid-membrane on breaks in helices, forming striking flexible central hydrophilic axis of the MD, extending from the Q cavity to MD tip^5,16^.

The major question in the complex I field is how the redox energy of NADH:UQ reaction, happening almost exclusively in the PA is coupled, over distances of up to 150 Å, to proton translocation in the membrane domain. Our current model of the complex I action assumes an existence of a structural/conformational mechanism, which would couple the reduction/protonation of UQ to the processes of proton transport mostly via the central hydrophilic axis^5,16^. Available structural information and results of molecular dynamics simulations suggest that this mechanism might indeed rely on conformational changes propagating through the membrane domain^5,22,23^. However, despite intense studies^24–27^, the existence of such a path has not yet been shown, mainly due to the lack of atomic resolution data on complex I in different redox/conformational states. Our model is not in disagreement with the “electrostatic spring” mechanism^28^. In the detailed model of such mechanism, a wave of electrostatic signals sequentially breaks lysine–glutamate ion pairs along the hydrophilic axis, leading to proton pumping^29^. In the latter model, however, the electrostatic interactions, and not conformational changes, are hypothesized to dominate the mechanism from quinone reduction onwards.

In this work we address this question by building and analyzing atomic structures of nine different conformational states of complex I, which were obtained by X-ray crystallography and single-particle cryo-EM. This includes four cryo-EM structures of complex I in the presence of NADH or NAD^+^ without any Q-site ligand, a crystal structure with bound NADH without Q-site ligand (CXI_NADH_), a crystal structure with decylubiquinone (DQ) bound in Q-site (CXI_DQ_), and another three structures with the Q-site-bound inhibitors, piericidin A (CXI_PIE_), aureothin (CXI_AUT_), and pyridaben (CXI_PYR_). Preliminary information on the mode of binding of DQ and piericidin A was reported^16^, however at that time the models were not fully refined and so were not deposited in the PDB.

The addition of NADH to complex I would lead to the reduction of the entire FeS chain (approximately every other cluster in the chain, due to interactions between the clusters^30^), including the terminal cluster N2 near the Q site^31^. Therefore, we expected that reduction by NADH in itself may lead to significant large-scale conformational changes. However, we did not observe such changes in our studies. In contrast, binding of DQ substrate to Q-site, in the absence of NADH or NAD^+^ in the NADH-binding site, promotes the rotation and tilt of the entire PA, including subunits Nqo4 and Nqo6 near Q-site, correlated with concerted changes in the region of the nearest proton pump, the E-channel (Fig. 1). This suggests that motions near the Q-site can lead to changes in the E-channel and can be driven just by DQ binding, without any redox reaction. Propagation of conformational changes further into the antiporter-like subunits possibly requires a full catalytic cycle. Our studies also highlight the importance of a junction region between the PA and MD, which transmits conformational changes from the Q-site to E-channel, coupling complex I into one functional entity. Overall, our results show a key role of quinone in the coupling mechanism of complex I.

## Results

### Conformational heterogeneity of complex I is not induced by NAD(H)

**Fig. 2:**
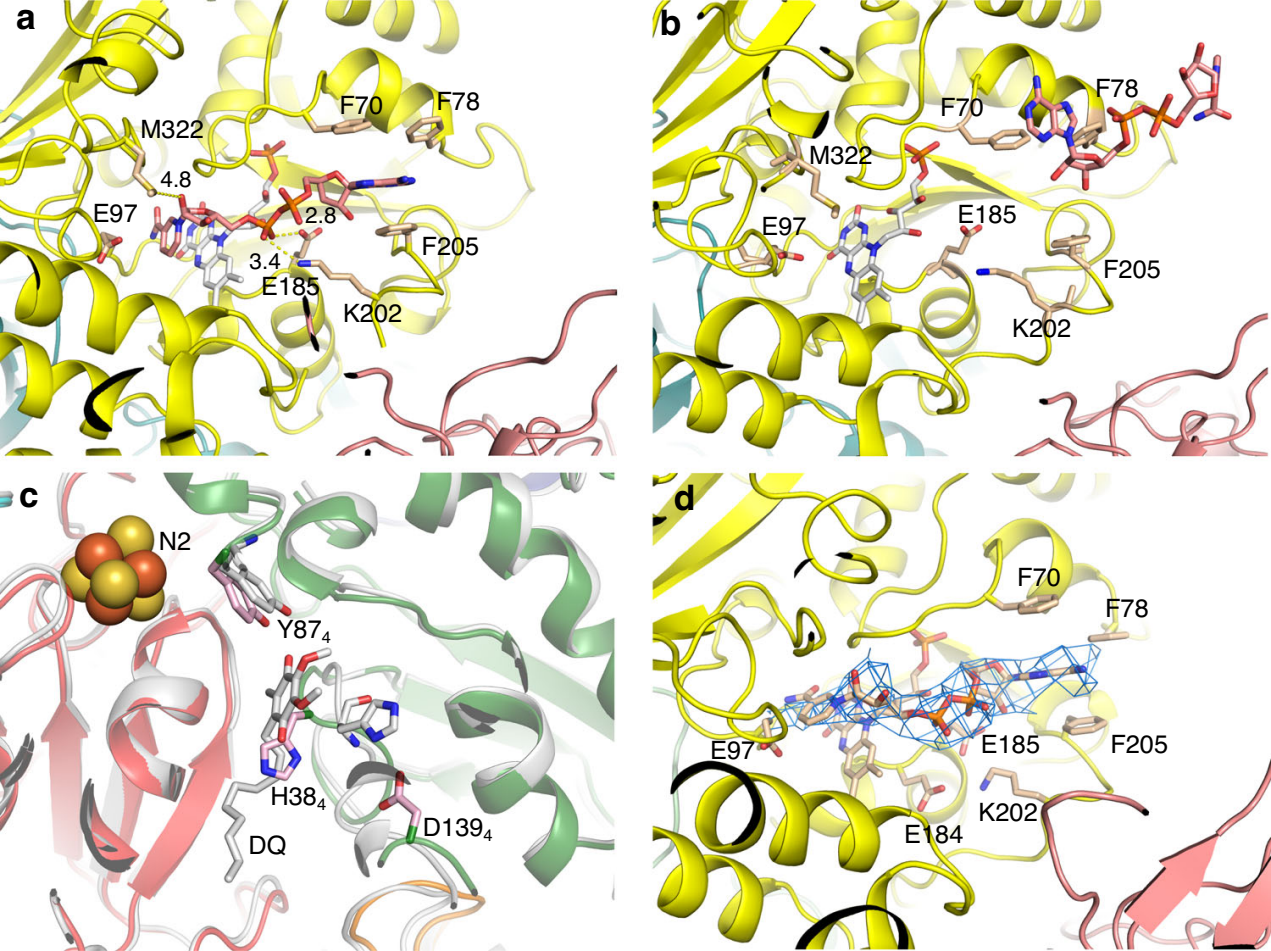
NADH-binding site in complex I. **a** The main interactions of NADH with subunit Nqo1 (CXI_NADH_ structure, after short soaking). FMN is coloured in white, NADH is in salmon. Key Nqo1 residues are labelled and the main NADH–protein interactions are indicated with yellow dashes, with distances in Å. **b** NADH binding out of its pocket after 5 h of soaking, ~15 Å away from FMN. **c** A structure obtained after overnight soaking with NADH shows a flip of H38_4_, key residue involved in the reduction of Q at the Q-site. The CXI_DQ_ structure is overlaid and coloured in grey, illustrating potential clash of DQ with flipped H38_4_. **d** Cryo-EM map section demonstrating that NADH is bound to complex I in the CXI_MJ_:NADH structure.

In this section, we discuss three sets of experiments: soaking of complex I crystals in NADH and cryo-EM reconstructions of complex I incubated either with NADH or with NAD^+^ (Fig. 2). The initial rationale for these experiments was the expectation that a full reduction of the complex might shift the structure into alternative conformation, linked to the coupling mechanism. Also, previously we observed that the exposure of crystals of the isolated PA of the complex to NADH led to shifts of some helices in Nqo4/6 subunits^32^. Crystallization efficiency and yield were dramatically improved by adding heterologously expressed subunit Nqo16 to the purified complex I (which contains sub-stoichiometric amounts of Nqo16). Our native structure of intact enzyme (PDB 4HEA [10.2210/pdb4HEA/pdb]) was updated (CXI_INT_,PDB 6Y11 [10.2210/pdb6Y11/pdb]) by re-building one loop near the Q cavity (residues 55–70 of Nqo6) according to the improved density from the 11 merged isomorphous native datasets and by refinement against this higher resolution (3.1 Å) data (Supplementary Table 1). This loop had a rather weak density previously and in the rebuilt conformation it is consistent with mammalian complex I structures^17^.

**Fig. 3:**
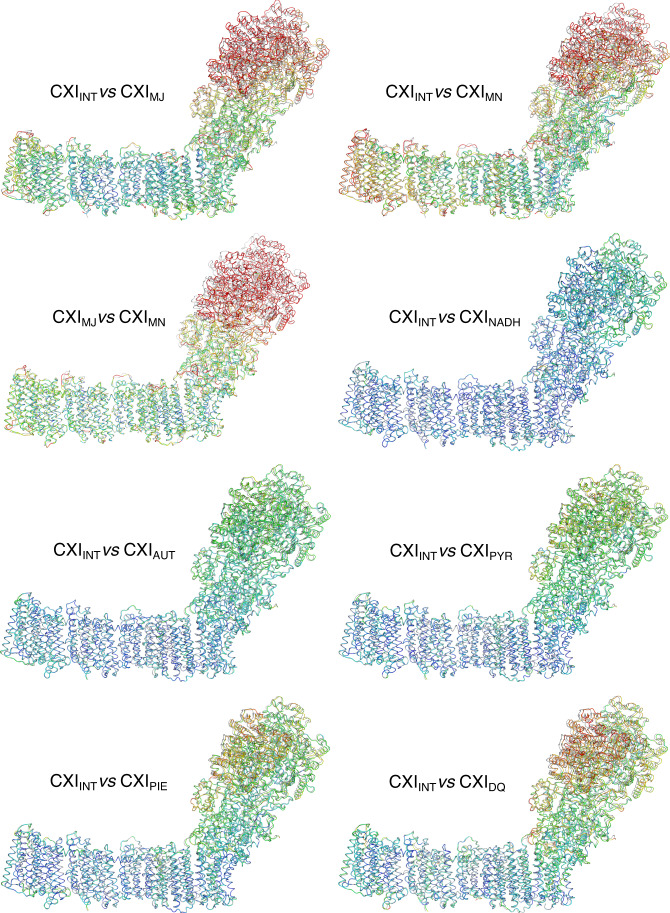
Different conformations of complex I. Models were aligned by Cα atoms of the membrane domain and coloured by Cα RMSD. Structure of native CXI_INT_ state is in grey. RMSD values range from 0 Å (blue) to 3 Å (red) or higher. Cryo-EM structures show significant PA rotation. Among the crystal structures, the DQ-bound structure (bottom right) shows the most significant conformational changes.

Our crystal structure of intact complex I with bound NADH confirms the binding mode of the dinucleotide observed previously with isolated PA^32^. The FMN is coordinated by subunit Nqo1 at the deep end of a solvent-exposed cavity that contains also the NADH-binding site. The binding of NADH relies on stacking and polar interactions (Fig. 2a)^32^. The nicotinamide ring is located in the inner region of the pocket, establishing a stacking interaction with FMN flavin ring while polar contacts with charged residues K202_1_ (suffix indicates subunit) and E185_1_ stabilize the diphosphate moiety. The adenosine ring is stabilized by stacking interactions with F70_1_ and F205_1_. Binding of NADH per se had no major effect on the structure of complex I, as the crystal structure of the CXI_NADH_ shows a very high level of structural similarity to the crystal structure of the native state, CXI_INT._ This is clear from per-residue RMSD values indicated on the structure (Fig. 3), and by RMSD values obtained by global superposition of the compared structures (Table 1). However, this is true only after relatively short incubation times with NADH of up to about 30 min. Since at least three NADH oxidation cycles are required to fully reduce the redox chain in complex I^33^ and the rate of NADH diffusion into crystals during soaking is limited (probably at least to the scale of minutes), it is possible that these structures represent a partially reduced complex, where NADH is bound, but FMN and some of the reducible clusters in the chain are still oxidized.

**Table 1:** The RMSD values for the aligned structures of complex I.

Structures of complex I aligned by C_α_ atoms of their membrane domains. CX, CX_MD_ and CX_PA_ stand for the whole complex, membrane domain and peripheral arm, respectively.						
Reference: CXI_INT_						
CXI_MJ:NAD+_	CXI_MJ:NADH_	CXI_MN:NAD+_	CXI_MN:NADH_	–	–	–
CX-2.00	CX-2.19	CX-2.88	CX-2.71			
CX_MD_-1.16	CX_MD_-1.12	CX_MD_-1.33	CX_MD_-1.33			
CX_PA_-2.54	CX_PA_-2.84	CX_PA_-3.77	CX_PA_-3.53	–	–	–
Reference: CXI_INT_						
CXI_NADH_	CXI_AUT_	CXI_PYR_	CXI_PIE_	CXI_DQ_	CXI_MJ_	CXI_MN_
CX-0.56	CX-0.83	CX-0.95	CX-1.06	CX-1.32	CX-2.00	CX-2.88
CX_MD_-0.43	CX_MD_-0.53	CX_MD_-0.54	CX_MD_-0.60	CX_MD_-0.73	CX_MD_-1.16	CX_MD_-1.33
CX_PA_-0.66	CX_PA_-1.02	CX_PA_-1.21	CX_PA_-1.35	CX_PA_-1.68	CXI_PA_-2.54	CX_PA_-3.77
Reference: CXI_MJ_						
–	CXI_AUT_	CXI_PYR_	CXI_PIE_	CXI_DQ_	–	CXI_MN_
	CX-2.32	CX-2.62	CX-2.75	CX-2.80		CX-2.43
	CX_MD_-1.14	CX_MD_-1.280	CX_MD_-1.31	CX_MD_-1.24		CX_MD_-1.19
–	CX_PA_-3.02	CX_PA_-3.42	CX_PA_-3.59	CX_PA_-3.69	–	CX_PA_-3.17
Reference: CXI_MN_						
–	CXI_AUT_	CXI_PYR_	CXI_PIE_	CXI_DQ_	–	–
	CX-2.88	CX-3.17	CX-3.13	CX-2.95		
	CX_MD_-1.27	CX_MD_-1.41	CX_MD_-1.44	CX_MD_-1.36		
**–**	CX_PA_-3.80	CX_PA_-4.18	CX_PA_-4.12	CX_PA_-3.87	–	**–**

Upon prolonged crystal soaks (from ~5 to 24 h, enough time for NADH solution to exchange several times in crystals) it is likely that the complex, including FMN, is fully reduced. In this case we observe two local changes in the structure, while globally it is still similar to the intact state: first, NADH does not bind in its usual position but rather at the periphery of the site (Fig. 2b) and the density for FMN is very weak; second, key H38_4_ from the β1 to β2 loop in Nqo4, which normally coordinates Q in the Q-site near cluster N2, flips over, clashing with the usual Q headgroup position (Fig. 2c). These changes probably reflect the reduced states of FMN and N2, respectively, and may help regulate complex I activity, as reduced FMN will prevent NADH binding until the redox chain is oxidized and ready to accept electrons, while reduced N2 (and probably also nearby cluster N6a) may help eject reduced quinol by promoting H38_4_ flipping and pushing Q headgroup out. A similar flipping out of H38_4_, but with more extensive loop movements, also leading to a possible ejection of quinone, was proposed previously on the basis of MD simulations^34^.

H38_4_ interacts with key conserved D139_4_, sitting on a C-terminus of a helix forming a prominent 4-helix bunch in Nqo4. In all our structures these residues are close enough (~2.5–3.0 Å) to form an ion pair (Supplementary Fig. 8a), and only in the long NADH soak structure the flipped out H38_4_ moves about 6 Å away from D139_4_, suggesting that D139_4_ probably became neutral.

We did not observe shifts of Nqo4/6 helices or a disconnection of one of coordinating cysteines from cluster N2 as was the case upon soaking crystals of isolated PA in NADH^32^. The likely reason is that upon removal of MD, the subunits from PA/MD interface in the isolated PA are somewhat de-stabilized and can undergo larger conformational changes than within intact enzyme. Soaking of crystals with NAD^+^ did not lead to any visible density in the FMN pocket, probably because of the very low affinity of oxidized complex I for NAD^+^ (~1–2 mM)^35^. Correspondingly, structures of complex I soaked with NAD^+^ are identical to CXI_INT_ and serve mainly as a control.

Cryo-EM analysis of complex I incubated in the presence of either NADH (dataset 1) or NAD^+^ (dataset 2) in the solution (before plunge-freezing) yielded two 3D classes differing in the overall conformation, the so-called major state (~60% of particles) and a minor state (~40% of particles). The conformation of each class and the distribution of contributing particles in each dataset (Supplementary Figs. 3–5) did not depend on the presence of NAD^+^ or NADH. NAD^+^ is not expected to have any effects on enzyme conformation due to low affinity but it was added to allow direct comparison with NADH-bound state. Cryo-EM density for bound NADH was clearly observed in maps from NADH data (Fig. 2d) and a weak density for the adenosine part of nucleotide was observed in NAD^+^ major state. Major states are at a higher resolution (~4.2−4.3 Å) due to the larger total amount of particles than in minor states (at ~6.0 Å resolution). Both conformers of the major class (CXI_MJ:NADH_ and CXI_MJ:NAD+_ states) show almost identical RMSD of 2.00 and 2.19 Å when superposed on the CXI_INT_ crystal structure. The same applies to minor states, which show RMSD of 2.88 and 2.71 Å for NAD^+^ and NADH, respectively. Membrane domains of both major and minor states, superposed on the CXI_INT_ crystal structure, align particularly well (Table 1), which indicates that the observed differences in the RMSDs are mainly due to the structural differences in the PA region. This, in turn, suggests that Q-site-free complex I can freely oscillate between two different conformational states (major and minor), which differ in the orientation of PA. In our conditions, the NADH cryo-EM sample should have been fully reduced, however, the density for the key β1–β2 loop in Nqo4 is not sufficiently resolved in the major classes, so we could not confirm whether H38_4_ flips, as would be expected, in this case. For simplicity, in the remaining part of this paper, we will refer to these states as CXI_MJ_ (major) and CXI_MN_ (minor), respectively, and no distinction will be made between NADH/NAD^+^ conformers of the same class. The distribution of particles indicates that major state is energetically more favourable than minor, and this does not change upon complex I reduction by NADH.

Cross-comparison of the CXI_MJ_ and CXI_MN_ states revealed a pronounced hinge-bending motion of PA in the CXI_MN_ state, which produced an overall more open conformation (a larger angle between the two arms) of CXI (Fig. 3, Supplementary Fig. 7b). We also observed local structural changes, distributed evenly throughout the compared structures (Supplementary Fig. 1). However, limited resolution of the minor state does not allow us to speculate about the possible functional importance of these changes. As cryo-EM structures are DQ-free, we compared these first to the crystal structure of the intact state. Structures of CXI_INT_ and CXI_MJ_ states were very similar at both global and local levels (Fig. 3 and Table 1, Supplementary Fig. 1), differing mainly in a slight rotation of PA in the CXI_MJ_ state (Supplementary Fig. 7c). With the exception of this rotation, both structures share a high degree of structural conservation, which suggests that in practice both of them represent the same conformational state of CXI. The observed differences may be mainly due to the crystal contacts, possibly restraining motion of PA in the CXI_INT_ crystal structure. On the other hand, the structure of CXI_MN_ state shows larger differences to CXI_INT_ state. This relates to both a larger motion of PA and even more pronounced differences in the region of E-channel (Supplementary Figs. 1 and 7c). Overall, CXI_MJ_ cryo-EM state is similar to CXI_INT_ crystal structure, while CXI_MN_ state is more similar to CXI_DQ_ crystal structure, described below, as can be seen from the similarity in INT–DQ and MJ–MN transitions (Supplementary Fig. 7a, b).

### Binding of DQ induces a pathway of local conformational changes

**Fig. 4:**
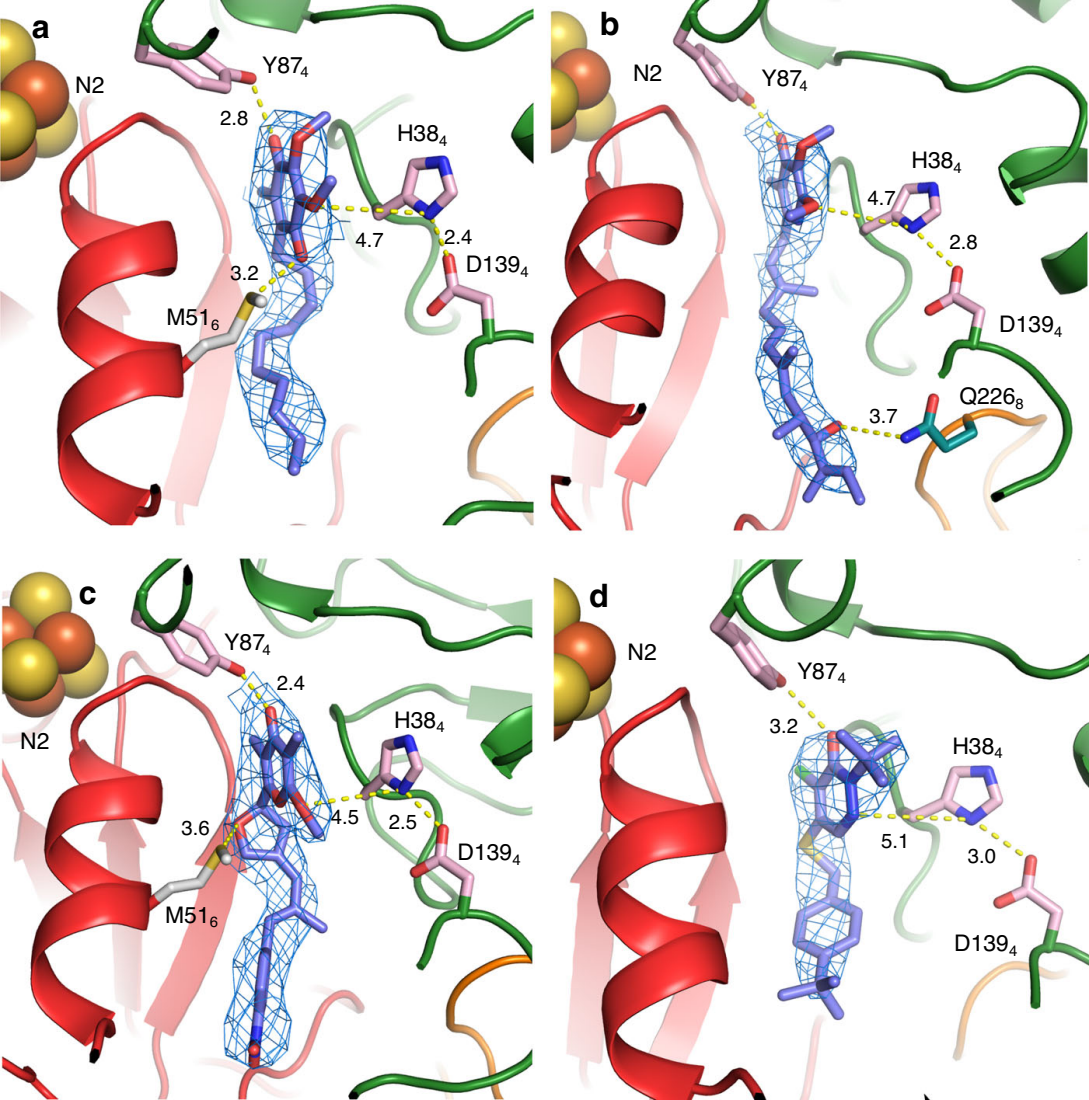
Structures of ligands bound in the Q-site. 2Fo−Fc maps (at 1*σ*) and main interactions are shown, with distances in Å. Nqo4 is coloured in green, Nqo6 is in red, and Nqo8 loop is in orange. **a** Decylubiquinone, **b** piericidin A, **c** aureothin and **d** pyridaben.

Our preparation of *T. thermophilus* complex I shows a high activity (similar to that in the membrane) with DQ of about 40 μmol NADH min^−1^ mg^−1^, which is 100% inhibited by rotenone or piericidin A, confirming that the complex is fully active and intact. IC_50_’s for a range of common complex I inhibitors are listed in the Supplementary Table 3. Aureothin, pyridaben and piericidin A are the most potent inhibitors of *T. thermophilus* complex I, while rotenone is less effective than it is with mammalian enzyme. Crystal structures of complex I with bound DQ and Q-like inhibitors also show a rotation of PA, like that observed in MJ–MN transition. However, its degree of torsion depends strongly on the Q-ligand, with DQ introducing the largest conformational change (Supplementary Fig. 7d, e). All Q-like ligands studied here display a similar pattern of interactions at the deepest end of the Q-cavity, with the headgroup about 12 Å away from cluster N2 (Fig. 4). A shared feature between all ligands bound in the Q-site is a hydrogen bond interaction of a ketone group from the quinone ring with the conserved and essential for complex I activity Y87_4_^16,36^. Other key residues, such as H38_4_ from the key β1–β2 loop (33-QHPSTHG-39) in Nqo4 and M51_6_, also contribute to the stabilization of the quinone ring, while secondary residues are involved specifically in the binding of each ligand (Fig. 4 and Supplementary Fig. 2). In particular, DQ interacts mainly with the residues mentioned above with its hydrocarbon chain surrounded by hydrophobic residues that complete the stabilization of the entire ligand (Fig. 4a). Piericidin A shows similar interactions with the addition of a hydrogen bond between Q226_8_ and the distal carbonyl group of its tail (Fig. 4b). In the case of aureothin, H38_4_ stabilizes its outer ether group, while M51_6_ interacts with the oxygen in the furanose ring (Fig. 4c). The distal nitrobenzene moiety is surrounded by both charged and nonpolar residues that, despite the lack of direct interaction, might form a suitable pocket for its accommodation. Finally, pyridaben seems to be stabilized only through the conserved interaction with Y87_4_ (Fig. 4d), although its proximity to Q33_4_ (3–4 Å) could facilitate an interaction with both the thioether and chloride groups. In addition, the tert-butyl group points towards a small hydrophobic cavity and the 4-tert-butylcyclohexyl moiety is surrounded by non-polar residues, completing its accommodation. In these structures quinone-like compounds are bound in the main “deep” site within the cavity, where quinone can effectively accept electrons from cluster N2. In structures from other species quinone was observed further away from N2, at intermediate sites on approach to the “deep” site^25,37^.

**Fig. 5:**
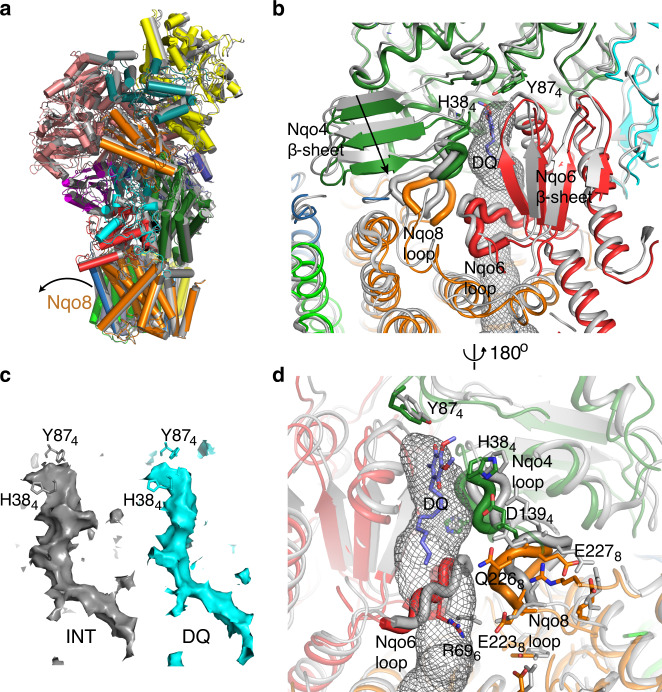
Conformational changes observed after binding of DQ. **a** Overlay of CXI_INT_ (grey) with CXI_DQ_ structure (coloured subunits, Nqo4 in dark green, Nqo6 in red and Nqo8 in orange). The structures were aligned by the MDs. Note that upon DQ binding Nqo8 tilts in the direction of the arrow, with tilt developing further in the PA. **b**, **d** Zoom-in into the Q-site, the same alignment as in **a**, with CXI_INT_ in grey and CXI_DQ_ coloured. The Q cavity is indicated with a grey mesh (calculated in MOLE) and bound DQ is in violet. The movement of the key Nqo4 β-sheet is indicated by an arrow. Key loops flanking the Q-site (Nqo4 32–39, Nqo6 66–75 and Nqo8 223–229 residues) are shown as thick tubes, with key residues from these loops indicated. **c** The Q cavity shown as solvent-accessible surface (calculated in Pymol with 1.4 Å probe). Note that the cavity gets narrower around H38_4_ residue in the CXI_DQ_ structure (cyan) as compared with CXI_INT_ (grey).

**Fig. 6:**
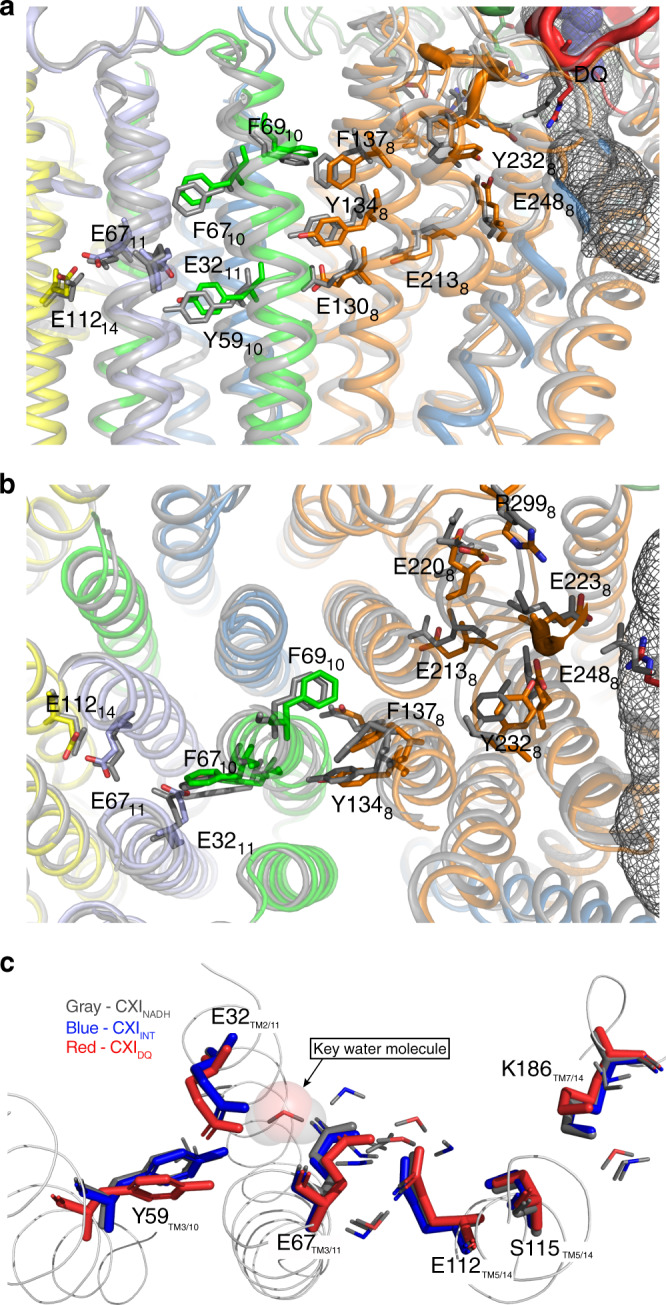
Propagation of conformational changes from the Q-site towards E-channel. **a**, **b** Overlay of CXI_INT_ (grey) with CXI_DQ_ structure (coloured subunits, Nqo8 in orange, Nqo10 in light green, Nqo11 in light blue and Nqo14 in yellow). The structures were aligned by the MDs. The distances between the key residues from the E-channel and the central hydrophilic axis, involved in proton translocation, change due to both global and local shifts and rotations of the side chains. A grey mesh indicates Q cavity. Key charged residues from the E-channel and hydrophobic residues undergoing large conformational changes are indicated. Y59_10_ sits on a π-bulge in the key TM3 of Nqo10. **a** Side view and **b** top view from the cytoplasm. The conformational change extends from the Nqo8 loop (red) towards Nqo11, coupled with major shifts in the hydrophobic protein environment. **c** Analysis of modelled water molecules distribution. In the crystal structure CXI_DQ_, rotation of the Nqo10 TM3 helix and the associated Y59_10_ leads to subsequent rotation of E32_11_, which allows for inclusion of a single water molecule between E32_11_ and E67_11_. The key water in CXI_DQ_ system is shown as transparent van der Waals spheres. All other modelled waters are shown as sticks with oxygen coloured according to the structure and hydrogen grey.

The binding of these ligands allowed us to identify the existence of a pathway of local conformational changes (Figs. 5 and 6), which propagate from the Q-cavity towards the proton pumps through Nqo4, Nqo8, Nqo10 and Nqo11. Most dramatic changes are caused by DQ binding, whilst Q-like inhibitors alter complex I conformation consistently in a similar way but to a lesser degree, to states intermediate between CXI_INT_ and CXI_DQ_ (Supplementary Fig. 7d). The overlap analysis (OA) (technique described below) shows that transitions from CXI_INT_ structure to CXI_AUT_ and CXI_PYR_ involve relatively minor PA rotation component (mode 8), which is much stronger for CXI_PIE_ structure and is accompanied in addition by a bending motion (mode 7) for CXI_DQ_ structure (Supplementary Fig. 7e). Structural alignments between individual subunits of CXI reveal significant local conformational changes in an area covering about 30 Å around the Q cavity, involving mainly residues of Nqo4 (key β1–β2 loop and D139_4_) and Nqo8 (Q226_8_ and E227_8_ from the key Nqo8 TM5–6 loop lining Q cavity, and R299_8_), which were implicated previously in coupling the quinone reduction with the proton pumps^5,16,22^.

The re-rearrangements in the Q-site after the binding of a ligand involve also more global shifts, which are revealed when structures are aligned by MDs. They originate from the significant tilting of TM helices of Nqo8, which develop further into PA tilt and rotation (Fig. 5a). Within Nqo4 and Nqo6, these changes are not limited to loops lining Q cavity but also involve two key β-sheets (Nqo4 residues 26–59 and the main Nqo6 β-sheet) that flank the cavity and undergo overall shifts of up to 4 Å (calculated for structures aligned by MD) (Fig. 5b). These β-sheets form a base on which PA is attached to MD and so, together with tilting TM helices from Nqo8, their movements likely are responsible for PA bending and rotation in response to ligand binding. The presence of DQ in the Q-site significantly alters the conformation of the Nqo4 β-sheet, pulling β1–β2 loop (residues 32–39) about 1.5 Å inside the cavity towards DQ, allowing for stacking interactions between H38_4_ and DQ (Fig. 5b, d). The opposite Nqo6 β-sheet also shifts in a similar direction, but the strand flanking the Q-cavity (P38_6_ to F41_6_) remains in place, thus effectively moving into the cavity and narrowing it. Connected to this β-sheet, Nqo6 loop R66–V75 interacts closely with the key Nqo8 TM5–6 loop (residues 223–229) (Fig. 5b, d). Both loops extensively line the Q cavity and interact via conserved charged residues. Therefore, as a result of DQ binding, the entire Q cavity undergoes conformational changes so that it “tightens” significantly around the quinone headgroup (Fig. 5c). According to CASTp 3.0 calculations^38^ with a 1.4 Å probe, upon DQ binding the surface area inside the cavity decreases from 1703 to 1297 Å^2^ and the enclosed volume decreases from 997 to 812 Å^3^.

The Nqo4 β1–β2 loop strongly pulls on the Nqo4 helix α4 through the interaction of H38_4_ with D139_4_, causing this helix to push on Nqo8 TM5–6 loop (Fig. 5b, d). As a consequence, Q226_8_ experiences a shift of ~3 Å into the Q cavity. The shift of this Nqo8 loop is probably the reason for the overall tilt of Nqo8 TM helices, which is most significant near the interface with PA. This distortion reaches Nqo8 TM3–4, where the hydrophobic region (V133_8_– W141_8_ and L149_8_–L153_8_) is in contact with the hydrophobic region of Nqo10 TM3 (A60_10_–I71_10_), next to the key Y59_10_ (Fig. 6a, b). That way conformational changes propagate deep into the E-channel and towards the first antiporter-like subunit.

Nqo10 TM3 is the most highly conserved helix in the MD and is critical for activity^5,16^. The entire cytoplasmic half of this helix, including F67_10_, rotates about 30° (Fig. 6b and Supplementary Fig. 6). The extent of this motion is much smaller than in mammalian complex I, where the same half-helix rotates almost 180°^21,39^, but the overall tendency is the same. Although it was proposed that in mammals such an extreme rotation is part of Active/Deactive transition^21^, our data strongly suggests that it is rather a part of catalytic cycle transitions between “open” and “closed” states of the complex, and the Deactive state is a more unfolded version of the “open” state^39^. Overall, the re-arrangements of the key loops around Q site in mammals are much more extensive than in *Thermus* (Supplementary Fig. 8). While in *Thermus* the key Nqo4, Nqo6 and Nqo8 loops move mostly as rigid bodies (Fig. 5), in mammals these loops can undergo significant re-arrangements^21,37,39^ (Supplementary Fig. 8). Nevertheless, the ground-state structures (ovine closed or mouse active) are similar to CXI_INT_ and the tendencies upon transition into another state (ovine open or mouse deactive) are also similar (flipping out of the Nqo4 loop or of the first strand of the Nqo6 β-sheet) (Supplementary Fig. 8). Therefore, it appears that in general the conformational changes in different species have a similar pattern around Q site and Nqo10 TM3, but their extent is much larger in mammals, probably allowing for a more robust mechanism as compared to the bacterial enzyme.

In Nqo11, I25_11_–E32_11_ region undergoes a slight shift so that E32_11_ moves about 1.2 Å away from E67_11_, which in its turn approaches closer to E112_14_, a key residue of the second proton pump (antiporter-like subunit Nqo14, nearest to E-channel) (Fig. 6c). As a consequence, the interface between Nqo11 and Nqo14 in the area between E67_11_ and E112_14_ narrows, possibly facilitating inter-subunit proton transfer^5^. The p*K*_a_’s of the key E112 and K186 in Nqo14 will likely change as a result of these shifts, possibly helping proton transfer in the N-terminal half-channel of Nqo14, although the exact sequence of the events remains to be established. These observations correlate with previous site-directed mutagenesis results (summarized previosuly^15,16,40^) and molecular dynamics simulations, indicating that these residues might be involved in proton transfer^23,41^.

Therefore, overall movements of PA relative to MD, which we observe in various structures here and also in mammalian complex I^39^ appear to be linked to (and to be secondary to) re-arrangements around the Q-site. This is clear from comparisons of CXI_INT_ and CXI_DQ_ structures at the global (PA movements) and local (Q-site and E-channel) level as discussed above. The transitions between different states may be driven thermally (cryo-EM major and minor states) or can also be driven (or thermal equilibrium shifted) by the binding of ligand in the Q-site (crystal structures). The overall path of the observed conformational changes agrees with the previously postulated mode of action for complex I with the central hydrophilic axis in the MD playing a key role^5,16^. However, here the “wave” of conformational changes apparently stops at the E-channel and does not reach the antiporter-like subunits. This is possibly because the redox reaction (Q reduction and protonation) is not taking place in the explored conditions.

Additionally, the degree of conformational changes could be intensified in vivo due to binding of the long-tailed natural substrate menaquinone-8, which can form more extensive interactions within the Q-site. Importantly, as mentioned above, most regions around the Q-site show marginal changes in the CXI_NADH_ structure, with the exception of the key H38_4_ flipping after extended incubation with NADH. Overall, it is clear that the binding of ligand in the Q-site drives most of the global changes (i.e. in the cavity and beyond) while the reduction of the complex leads to more local re-arrangement of the Nqo4 β1–β2 loop (probably due to interactions with the reduced N2 cluster).

### Normal mode analysis (NMA)

**Fig. 7:**
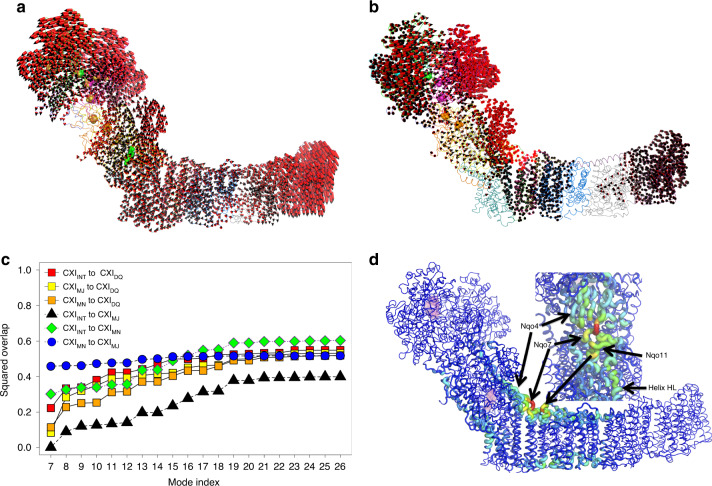
The results of normal mode analysis (NMA). Both lowest-frequency modes, mode 7 **a** and mode 8 **b**, bending and rotation respectively, are observed experimentally. The overlap analysis **c** shows that transitions to CXI_DQ_ conformation involve a large contribution of rotational motions (mostly mode 8 plus some additional modes), which is not the case for transitions between unliganded conformations, the CXI_INT_ → CXI_MN_ and CXI_MN_ → CXI_MJ_. The CXI_INT_ → CXI_MJ_ transition features no bending motion (mode 7) at all, but residual, localized rotational motions of higher frequency. The results of deformation analysis **d** identify the most flexible parts of complex I: the long loop of the Nqo7 subunit at the junction between PA and MD, as well as part of Nqo12 traverse helix HL, both involved in almost all of the first 20 motions (modes), which characterize the essential dynamics of complex I. The inset shows the view from PA.

The NMA is routinely used in probing essential motions of large molecular machines to describe major conformational motions, and also to describe directionality of these motions with potential functional connotations^42^. Thus, we decided to perform NMA on all nine conformations of complex I. The NMA revealed the existence of two large-scale domain motions. Both are shown schematically in Fig. 7a, b. The first one, the mode 7 (the lowest-frequency mode), can be described as a scissor-like bending motion of PA and MD, while the mode 8 (the second of the lowest frequency modes), involves rotation of PA. Both motions were predicted in previous NMA studies of complex I^43^ but importantly, here both motions were observed experimentally, when comparing structures of cryo-EM states and CXI_INT_/CXI_DQ_ states.

Specific differences accompanying transitions between these conformations can be resolved by NMA. We performed the so-called OA^42^, which allowed us to describe patterns of motions underlying the transitions between different configurations (Fig. 7c). The OA clearly demonstrates that conformational transitions between unliganded conformations and Q-site-ligated conformations are governed by different combinations, as well as by different contributions of the observed modes of motions, compared to transitions between Q-site-free conformers of enzyme. First, all transitions from the three unliganded CXI_INT/MJ/MN_ conformations to CXI_DQ_ conformation are characterized by exceptionally large contribution of rotational (mode 8) PA motions (Fig. 7c), in particular, within the Q-site region of a complex. This suggests that binding of DQ (or Q-site substrate/inhibitor generally) induces multiple rotational motions of a Q-site region, and indeed, as outlined above, such motions were revealed after superposing crystal structures of CXI_INT_ and CXI_DQ_ states. Second, transitions between unliganded configurations involve only bending (mode 7) of PA (CXI_INT_ to CXI_MN_ and CXI_MN_ to CXI_MJ_) or some rotation (CXI_INT_ to CXI_MJ_) but to a lesser degree compared to Q-site-liganded vs. unliganded states (Fig. 6c). We observed that both modes 7 and 8 appear to introduce changes in the region of the first proton pump, the E-channel. However, as the amplitude of the motion in NMA can be misleading, it is difficult to speculate about the real scale of these changes. Nevertheless, our NMA studies provide another line of evidence that global bending/twisting motions can be linked to local changes in the E-channel. Additionally, as shown by the OA (Fig. 7c), both modes have the largest contribution (compared to modes 9 and higher) to all studied conformational transitions, which stresses their overall importance for the function of complex I.

We also performed the so-called deformation analysis (on NMA data), which allowed us to identify hinge regions of complex I which enable these transitions (Fig. 6d). These are: a loop of the Nqo7 subunit, extending along the junction between the PA and the MD, the key Nqo4 β-sheet, as well as the C-terminus of the traverse Nqo12 helix. These elements couple motions of PA and MD, cementing the structure into a single functional entity. Finally, the OA yielded yet another interesting observation. None of the first 20 (Fig. 7c) or 50 lowest-energy modes even when combined together, suffice to fully describe the experimentally observed transitions (accounting for max ~60% of a given transition) between different conformers of complex I, suggesting that these also require multiple conformational changes of a more local character, e.g., loop motions or interactions between particular residues. This would agree with the postulated modus operandi for complex I^5,23^, where hopping of a proton must involve multiple atomic-level interactions between the residues involved.

### Allosteric interactions in complex I based on network analysis

The results so far (Figs. 5–7) suggest that motions around the Q-site and in the E-channel affect each other. We performed network analysis of correlated motions (CNA) in order to gain further insight into this issue (for technical details of CNA see “Methods” section). The CNA can be used to identify portions of proteins which are characterized by coupled dynamics and also to gain insight into the strength of this coupling^44^. In CNA, the portions of a protein which move as one coherent body are called communities (or nodes) and a possible coupling between communities is represented as a connecting edge. Thus, the system is being partitioned into the functional nodes based on a degree of collectivity of a motion, and the resulting networks, and hence partitioning of a system, have functional implications.

**Fig. 8:**
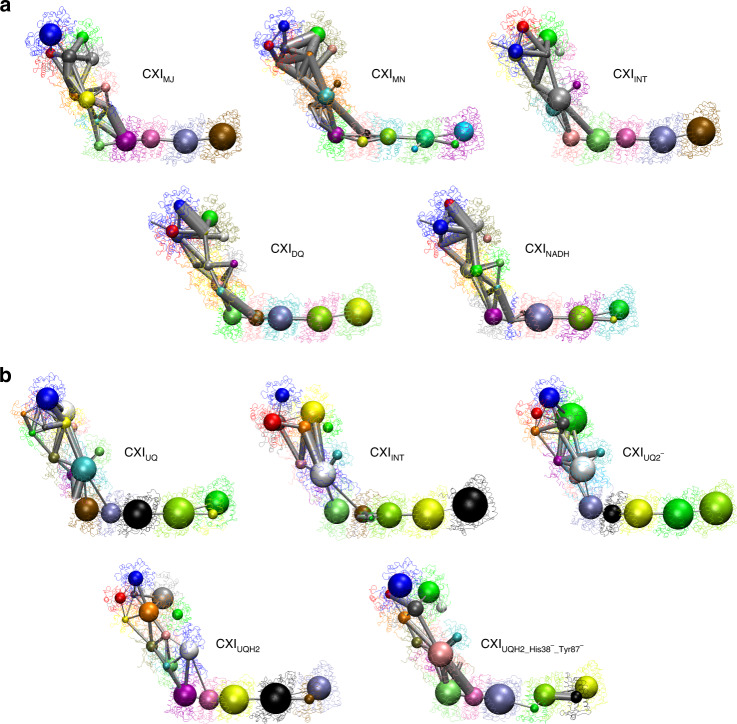
Network analysis of potential allosteric couplings between respective regions of complex I. **a** Analysis of experimentally determined structures, as indicated. **b** Analysis of the results of CG-MD simulations. Detected substructures or communities (nodes) of highly intra-connected (coupled) residues are shown as spheres, with sphere radius proportional to the number of residues included in the node. Colours of spheres correspond to particular communities, which vary between the systems. The grey lines or edges demonstrate sparse and functionally relevant connections (allosteric pathways of signal propagation) between these communities. The strength of coupling is proportional to the edge thickness. The NMA calculations were performed with the HCA force-filed and network analysis was performed with the correlation cut-off of 0.45 **a** or 0.50 **b**.

Calculated networks for all studied states of complex I show roughly similar partitioning (Fig. 8a). The upper portion of PA (Nqo1–3, NADH-oxidizing domain) shows a dense network of connections, suggesting coherent motions of this region as a single body. Further, while the coupling between the Nqo12–Nqo14 antiporters is very weak, it extends along their central portions (the hydrophilic axis^16^), suggesting that communication between channels might be correlated with the processes of proton transfer. Finally, all the constructed networks confirmed coupling between motions of the Q-site and the E-channel, or more precisely, between the Nqo4 subunit and the node comprising Nqo7/10/11 subunits. Strikingly, this coupling was the strongest in the CXI_DQ_ structure, because it was augmented by an additional edge, extending from the Nqo6 subunit (Fig. 8a). That is, motions of both Nqo4 and Nqo6 subunits appear to be coupled to the structural changes in the region of the E-channel, consistent with our experimental observations above.

### Coarse-grained molecular dynamics (CG-MD) simulations of complex I

The results of network analysis as well as the results of structural comparisons all suggest that re-arrangements near the Q-site are coupled to structural changes in the region of the E-channel. We performed CG-MD simulations in order to study this coupling in further detail. The simulations were performed in a coarse-grained representation using the Martini v.2.2 model^45^ for DOPC bilayer, protein, water, and ions (further details are in “Methods” section). The simulated systems included the CXI_INT_ state and several different Q-site-occupied configurations of CXI, structures of which are yet to be determined experimentally, that is, the CXI_UQ_, CXI_UQ2_^−^, CXI_UQH2_, and CXI_UQH2_H38_-__Y87_- states. Although *T. thermophilus* CXI uses menaquinone in vivo, in vitro it can equally well use UQ. Since we know from the CXI_DQ_ structure how the UQ headgroup binds to the complex, we used UQ for modelling. All the configurations contained UQ (with 10 isoprenoid units) at different stages of its reduction, which allowed us to investigate how the different redox forms of UQ might affect the mechanics of complex I, including the process of proton transport. The general equation of a reduction reaction is: UQ + 2e^−^ + 2H^+^ ↔ UQH_2_ (Fig. 5 in ref. ^46^). UQ first diffuses into the Q-cavity and forms hydrogen bonds with H38_4_ and Y87_4_. In our simulations, this state is represented by the CXI_UQ_ configuration. Next, UQ gets reduced (consecutively) by two electrons to ubiquinol dianion and this state is here simulated by the CXI_UQ__2_^−^ configuration. The CXI_UQH2_H38_-__Y87_- configuration represents a hypothetical stage when the negative charge gets temporarily stored on the putative proton donors H38_4_ and Y87_4_, as they donate a proton each to UQ2^−^. Finally, the CXI_UQH2_ configuration represents CXI with fully protonated ubiquinol, but before it leaves the Q-site.

Similarly to NMA, the trajectories produced by the simulations were used to construct networks of allosteric couplings, which were then plotted on the simulated structures of CXI. The obtained networks are shown in Fig. 8b and are in fact similar to the ones based on the results of NMA. These show a very strong coupling between Nqo1–3 domains, weakly coupled antiporters Nqo14, 13, and 12, and again, a clear correlation between motions of the Q-site (mainly Nqo4 subunit) and motions in the E-channel region. However, we also observed substantial differences between the constructed networks. The most striking observation was an exceptionally strong correlation between the motions of the Nqo4 subunit and the region of the E-channel in the CXI_UQH2_H38_-__Y87_- configuration (Fig. 8b), definitely the strongest among all the simulated configurations. This suggests that transfer of charge to the amino acids coordinating Q constitutes the strongest stimulus leading to changes in the E-channel. It is even stronger than the previously discussed rotation of the Q-site region. Strikingly, a reduction from UQ to double charged dianionic ubiquinol (to the CXI_UQ2_- state) does not suffice to induce such changes, as this configuration does not show any additional correlation between the Q-site and the E-channel (Fig. 8b). Finally, the structure of the CXI_INT_ state seems to be the only one which does not show a direct correlation between the Nqo8 subunit and the node comprising the Nqo7, 10, and 11 subunits. This suggests that the presence of the substrate in the Q-site (all configurations with the Q-site occupied) generally strengthens the coupling between the Nqo8 and the E-channel.

We further performed a more fine-grained analysis of the allosteric paths leading to these networks. The detailed paths have been calculated between H38_4_, coordinating quinone, and E112_14_, a key residue in the proton channel of the nearest antiporter-like subunit Nqo14, as shown in Supplementary Fig. 9 (with further details in Supplementary Data 1). Only the CXI_UQH2_H38_-__Y87_- configuration features the unique path between the Nqo4 β-sheet, the neighbouring helix and the E-channel. This unique path, passing close to the matrix side of the protein in the hinge region identified in Fig. 7d, can be attributed to the H38^−^/Y87^−^ charge effect, since other systems feature paths closer to the ones present already in the CXI_INT_ system. In all other configurations, even the one with charged UQ^2−^, the most frequently taken paths “bypass” the Nqo4 β-sheet (and helix), and go straight down through the Nqo8 Q-site loop. This is in agreement with the experimental findings, as the unique path is likely activated upon the observed rotation of PA and Nqo4 β-sheet. Summarizing, the results of MD simulations re-iterate our earlier findings and highlight a central role of charge transfer for the propagation of conformational changes from the Q-site to the E-channel.

### Water networks in complex I

As mentioned above, the membrane domains of all studied configurations align very well except for the E-channel, which displays a characteristic lack of structural conservation. Thus, we decided to solvate all the studied structures to identify any differences in the obtained solvation patterns. Previous MD studies hinted at a possible role of water networks in coupling between the Q-site and E-channel^47^. As the obtained resolution of cryo-EM states did not allow us to resolve orientation of side-chains of all residues implicated in proton transfer^16^, we focused mainly on the comparison of the solvation patterns in the crystal structures. We first determined protonation states of all the amino-acid residues using PROPKA^48^ and then solvated the structures using Dowser^49^. As expected, the structures show very similar water networks, extending from the Q-site to the E-channel and propagating along the MD central axis. The possible proton translocation pathways involving waters and Grotthuss-competent^50^ residues (Asp, Glu, Lys, His, Tyr, Ser, and Thr) were examined for each structure. These are not continuous across the membrane, as can be expected for a proton pump in order to prevent proton leaks. The links along the central axis are much more extensive, but include some breaks, for example in the E-channel between E130_8_ and Y59_10_. Possibly a link in this area may be established under turnover conditions. Further, as noted before^5^, the periplasmic side of antiporters is exceptionally dry, except for a distal antiporter Nqo12. Therefore, the exact output paths from the channels into the periplasm are currently not clear and require further investigation.

Despite the overall similarity of water networks, we identified one notable difference: only in the CXI_DQ_ structure we observe the presence of a single water molecule nested between E32 and E67 of Nqo11 subunit (Fig. 6c). In CXI_INT_ or CXI_NADH_ structures the distance between E32 and E67 is too large for efficient proton transfer (~3.7 Å). Thus, placement of water in this position leads to the formation of a unique continuous path extending from Nqo11 to Nqo14, that is, a path connecting the E-channel and the first antiporter. The inclusion of water is caused by the previously described cascade of changes propagating along the E-channel. The most striking manifestation of these changes is the rigid body rotation of the Nqo10 helix 3 (TM3_10_), and thus the rotation of its key residue Y59_10_, which flips out from the pocket between TM2 and TM3 of Nqo11 (TM2/TM3_11_) and gets closer to TM3_11_ (Fig. 6c). This allows for the subsequent rotation of the E32_11_ side-chain towards Y59_10_, which in turn leads to the formation of additional space between E32_11_ and E67_11_, allowing the inclusion of a water molecule. The movement of Y59_10_ is allowed by its position on the conserved π-bulge in TM3_10_. Therefore, our studies point to the rotation of key Y59_10_ as potentially having functional importance, allowing formation of a distinct functional network of water molecules, likely a prerequisite for proton transfer.

## Discussion

Complex I was found to fluctuate between the two different conformational states. The cryo-EM major and minor states observed in *T. thermophilus* may correspond to the “closed” and “open” states of mammalian complex I^39^, respectively, due to an “opening” movement of the PA in CXI_MN_ state compared to CXI_MJ_ (Fig. 7 and Supplementary Fig. 7). However, in contrast to *T. thermophilus*, in mammalian enzyme an “open” state comprises a majority of particles and the “opening” movement is much more pronounced^39^. The differences between open and closed classes in mammals also include much larger re-arrangements of the loops around the Q-site and an almost 180° degree rotation of TM3_10_ helix^39^ (Supplementary Fig. 8). Therefore, these differences are reminiscent of patterns that we observe in *T. thermophilus* but are much more dramatic. Similar but even more extreme re-arrangements (further unfolding of Q-site loops) may be involved in the Active/Deactive transitions of mammalian enzyme^21,51^. Since the conformational free energy landscape of complex I is characterized by the presence of at least two major and discernible minima in both mammalian and bacterial versions of the enzyme, it is also possible that the *T. thermophilus* CXI_MJ_ and CXI_MN_ states correspond to the mammalian “open” and “closed” states, respectively (according to states with a majority of particles). The exact correspondence of different classes between species remains to be established from high-resolution structural studies, allowing detailed comparisons of conformations of loops around the Q cavity. Nevertheless, it is clear that in the species studied so far complex I exists in at least two different global conformations, associated with changes around the Q cavity. This suggests that conclusions from our observations here are likely applicable to the entire complex I family.

In contrast to our original expectations, NADH binding and subsequent reduction of the enzyme does not lead to large conformational changes apart from flipping of H38_4_ upon extended soaking, which likely plays a role in regulating Q access into the deep end of the cavity. However, binding of ligands to the Q-site leads to much more profound changes. It has the potential to switch the overall conformation of the CXI_INT_ state resembling CXI_MJ_ into the CXI_DQ_ state which resembles CXI_MN_. That is, Q-site binding by DQ, and to some extent Q-like inhibitors studied here, appears to shift the conformational energy minimum towards the CXI_MN_-like state, allowing us to observe such conformations in crystals. Our data show that quinone binding leads to the tilt of Nqo8 TM helices and movements of the β-sheets and conserved loops flanking the Q-site, causing “tightening” of the Q cavity around the ligand. In turn, this leads to the rotation of the PA and to the structural changes along the path connecting the Q-site to the E-channel, causing translational/rotational motions in the Nqo7, 8, 10, and 11 subunits. The observed changes are of a moderate scale even in the CXI_DQ_ conformation and do not appear to extend beyond the E-channel. This indicates that the charges brought in by quinone redox reactions are probably needed to increase the strength of coupling between the PA and the E-channel and to initiate changes in the antiporter-like subunits. It remains to be established, from high-resolution turnover studies, whether the antiporters are driven mostly conformationally or via electrostatic interactions. Additionally, the presence of the lipid membrane and the pH gradient may be necessary in order to fully imitate in vivo conditions.

**Fig. 9:**
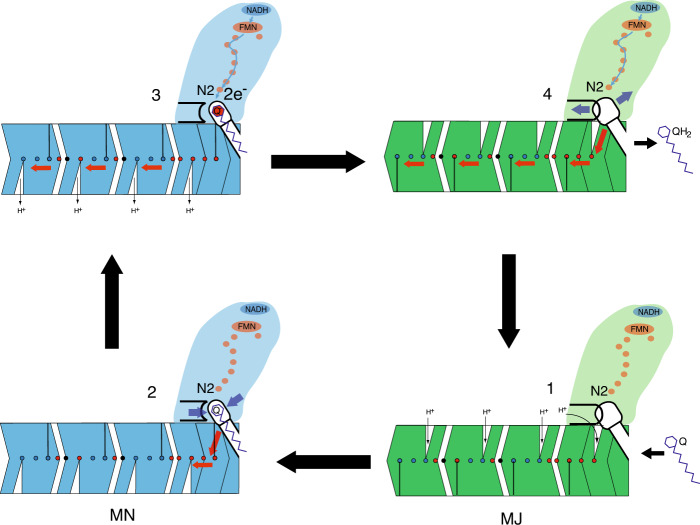
The coupling mechanism of complex I. The two main states of the complex are shown in green (major-like MJ states, represented by CXI_INT_, CXI_NADH_, and CXI_MJ_ structures) and blue (minor-like MN states, represented by CXI_DQ_ and CXI_MN_ structures). It is expected that in the membrane such two states would largely correspond to the proton channels opened either to the cytosol (MJ) or to the periplasm (MN). In state 1 the enzyme is waiting for quinone to bind. The complex is reduced so that key Nqo4 β1–β2 loop with H38 is flipped into the Q cavity (black arc). In state 2, upon quinone binding, this loop retracts, allowing quinone to reach the deep end of the cavity. The cavity “tightens” around the headgroup (blue arrows), the enzyme converts into the MN state and conformational changes propagate into the E-channel (red arrows). In state 3, two electrons from NADH arrive to quinone via cluster N2, the quinone is protonated to quinol and the double negative charge resides on proton-donating residues H38_4_ and Y87_4_ (depicted as red Q headgroup for simplicity). Electrostatic interactions with charged residues in the E-channel initiate further changes propagating (either conformationally or electrostatically) into antiporter-like subunits (red arrows), resulting in the ejection of protons into the periplasm. In state 4, the enzyme is quickly re-reduced and H38_4_ flips out (black arc). Facilitated by this and the expansion of the cavity (blue arrows), quinol is ejected into the lipid bilayer and the enzyme reverts to a MJ-like state. The cycle resumes at state 1, with further details outlined in the text.

On the basis of our results we propose a mechanism of action employing the global flexibility of complex I, which we see oscillating between at least two low-energy states, similar to CXI_MJ_ and CXI_MN_ (Fig. 9). These two different global conformations are probably associated with the switch in the exposure of proton channels to the different sides of the membrane (achieved under turnover conditions with membrane present, as noted above). The major state is likely to be more “relaxed” with multiple access paths open for protonation from the hydrated^5^ cytosol side while the higher energy minor state may be associated with paths opening for proton ejection through the dry periplasmic side (Fig. 1). Indeed, as noted above, in the native crystal structure (CXI_INT_), corresponding to the CXI_MJ_ state, we can identify many possible proton transfer pathways linking the MD core to the cytosol, while connections to the periplasm do not seem to exist in this state. These connections are not visible in CXI_DQ_ structure either, possibly due to the absence of turnover as noted above. As Fig. 7c shows, CXI_MJ_–CXI_MN_ transition involves mainly bending of the complex (mode 7), while CXI_INT_–CXI_DQ_ transition also involves in a large part such a transition, but is complemented by a significant rotational component (mode 8). Therefore a part of the necessary conformational change is easily accessible by thermal fluctuations (CXI_MJ_ vs. CXI_MN_), but another part is likely to be evoked by quinone binding, while further changes in the antiporters may require turnover and the presence of membrane.

During the catalytic cycle, the FeS redox chain is likely to be almost constantly reduced, as the NADH-site reactions are fast and the total reaction is limited by quinone turnover^35^. Therefore, we start the cycle with the reduced enzyme waiting for the quinone to bind (State 1 in Fig. 9). In this major-like state the Q cavity is relatively open and the key H38_4_ is flipped into the cavity (as in crystal structures after long soaks), having helped to eject quinol in the previous step. Proton channels are accessible from the cytosol, so that the key residues in the MD core are protonated. Then, binding of quinone causes H38_4_ to flip back and leads to the overall “tightening” of the Q site, which induces hinge motions shifting global conformation towards the minor-like state (State 2 in Fig. 9, structure CXI_DQ_). Local changes are propagated from the Q-cavity towards the E-channel, although this is not sufficient to switch the antiporter channels. As electrons are transferred from the NADH along the PA towards the quinone, it will be reduced and protonated (by H38_4_/Y87_4_) to QH_2_ (State 3 in Fig. 9). Negative charge on H38_4_/Y87_4_ will drive further conformational changes in the E-channel (Fig. 8b and Supplementary Fig. 9). The reaction will propagate further towards the antiporters either via conformational changes or electrostatic interactions, resulting in the full switch of channels and lowering of the p*K*_a_ of the terminal residues in each channel, so that protons are ejected into the periplasm. The presence of the lipid membrane may be necessary to achieve the full extent of conformational changes. Then, QH_2_ will lose the stability at the Q-site and, pushed by the flip of H38_4_, will migrate towards the exit from the cavity to join the quinone pool in the membrane. This will release Nqo4 and Nqo8 loops from the lock; they will return to their original positions along with the key residues in the E-channel, as well as allow re-protonation of H38_4_/Y87_4_, bringing us to State 4 in Fig. 9. The cycle resumes in State 1 as the enzyme gathers the few protons available from the cytoplasm in order to re-protonate the MD core. Overall, joint action of negative charges arriving to the Q-site and Q/QH_2_ movements in and out of cavity would result in proton translocation along the entire MD. The junction region between the PA and the MD plays a key role in this mechanism, by not only transmitting the signal from the PA to the E-channel, but also coupling the motions of both arms, and thus turning the structure of the complex into a functional molecular machine. Under this framework, quinone chemistry and associated conformational changes, rather than FeS clusters reduction, are the key to the mechanism of coupling in complex I.

## Methods

### Complex I purification

Intact complex I from *T. thermophilus* was purified through several steps. *T. thermophilus* HB8 cells were grown in a 70 L bioreactor system (Applikon Biotechnology, Delft, The Netherlands) at 70 °C, pH 7.4, for about 20 h. Membrane preparation and complex I purification was performed at room temperature from 150 g of these cells, firstly resuspended in 50 mM Bis–Tris pH 6.0, 0.002% (w/v) PMSF and EDTA-free protease inhibitor cocktail (Roche, Basel, Switzerland) and then solubilized with 2 mM CaCl_2_, 50 mM NaCl, and 1% *n*-tridecyl-β-maltoside (TDM, Glycon, Luckenwalde, Germany) for 1 h. After centrifugation at 150,000 × *g* for 1 h and filtration (0.45 μm), solubilized material was passed through a HiLoad 26/10 Q-Sepharose anion exchange column equilibrated with buffer A (20 mM Bis–Tris pH 6.0, 2 mM CaCl_2_, 0.002% (w/v) PMSF, 0.05% TDM, and 10% glycerol) and eluted with a linear gradient of 1 M NaCl in buffer B (1 M NaCl in buffer A). Eluted material was passed through a HiPrep 16/10 ANX FF anion exchange column equilibrated with buffer A. After elution with buffer B, fractions were pooled and diluted to about 5 mM NaCl in buffer A, then loaded onto a Mono-S HR 16/10 cation exchange column equilibrated with buffer A. Fractions were eluted in a linear gradient of 0.5 M NaCl in buffer A. All the fractions along the purification process were assessed by SDS–PAGE and NADH:FeCy activity. Fractions containing intact complex I were pooled and concentrated to about 1 mL (100 kDa cutoff MWCO), then loaded into a HiLoad 16/60 Superdex 200 gel-filtration column equilibrated with 100 mM NaCl in buffer A. Fractions with purity assessed by SDS–PAGE were pooled and concentrated to about 25 mg mL^−1^, stored in liquid nitrogen at 25% glycerol.

### Activity measurements

Complex I activity was measured at 50 °C with DQ as an electron acceptor. To a 2 mL quartz cuvette containing DQ assay buffer (50 mM Bis–Tris pH 6.0, 25 mM NaCl, 2 mM CaCl_2_), 0.25 mg mL^−1^ asolectin (Fluka), 8 μg mL^−1^ complex I protein, and 100 μM DQ were added. The sample was incubated for 5 min whilst stirred at 400 rpm, to allow for the equilibration of the protein/DQ/lipid/detergent mixture. The reaction was started by adding 100 μM NADH and the change in absorbance was followed at 340 nm, over 30 s under constant stirring. Specific enzyme activity was calculated in μmol NADH oxidized mg^−1^ ml^−1^. When required, inhibitors of complex I were added after DQ, prior to the 5-min incubation period. Lipids were prepared by mixing asolectin in a small volume of chloroform (1 mL) before evaporating the solvent under a stream of nitrogen gas, and were resuspended in 1% CHAPS to a final concentration of 5 mg mL^−1^ before use.

### Nqo16 purification

Subunit Nqo16 (*ttha1528* gene) was amplified from the *T. thermophilus* HB8 genome by PCR, using forward (5′-AATTAGCATATGGTACGCGTGGGCATGCGCGCC) and reverse (5′- AATTAGGGATCCCTAGGCCGCCCGCTTGAGGTAG) primers synthesized by Sigma Aldrich (Gillinghman, Dorset, UK). The products from the reaction were purified using a PCR purification kit (Qiagen, Hilden, Germany) and cloned into the pet16b vector, between NdeI and BamHI restriction sites, adding a polyhistidine tag to the 5′ end of the *ttha1528* gene. 1 μL of plasmid (50–100 ng μL^−1^) was added to a 100 μL aliquot of chemically competent *E. coli* XL1 blue cells, left on ice for 20 min then incubated at 42 °C for 45 s. 450 mL of S.O.C. medium was added to the transformed cells and incubated at 37 °C for 1 h. 50 μL of transformed cells were spread on an agar plate with ampicillin, allowing colonies to grow. The *ttha1528* insert was then validated by Sanger sequencing (Source Bioscience, Cambridge, UK). The plasmid pet16b containing *ttha1528* insert was extracted with a QIAprep Spin Miniprep Kit (Qiagen, Hilden, Germany) and transformed, as previously described, into *E. coli* BL21(DE3) cells, for further expression and purification.

One litre of LB containing 100 μg mL^−1^ ampicillin was inoculated with stocks of *E. coli* BL21(DE3) cells containing pet16b-*ttha1528* and grown in baffled flasks, without induction, for 18 h at 37 °C and 250 rpm. The cells were harvested by centrifugation (6800×*g* for 30 min, at 4 °C) and resuspended in 5 mL/g of cells of binding buffer (50 mM HEPES pH 7.4, 500 mM NaCl, 0.002% (w/v) PMSF and a PIC-E tablet), using a glass-Teflon homogenizer, at 4 °C. The cells were passed through a cell disrupter twice at 110 psi (SPCH-10, Stansted Cell Disrupter). The cell lysate was heat treated, in a water bath at 70 °C for 10 min, then clarified by ultracentrifugation (215,000×*g* for 1 h at 23 °C). The supernatant was retained and filtered through a 0.45 μm filter, then loaded onto a 5 mL Ni-NTA Superflow Cartridge (Qiagen, Hilden, Germany), pre-equilibrated with binding buffer. A gradient of elution buffer (binding buffer and 500 mM imidazole) was applied to elute the His-tagged Nqo16. Eluted fractions were monitored by *A*_280_ and analysed by SDS–PAGE. The buffer was exchanged into the storage buffer (20 mM Bis–Tris pH 6.0, 2 mM CaCl_2_, 100 mM NaCl, 0.05% (v/v) TDM and 25% (v/v) glycerol). Nqo16 was concentrated to 3 mg mL^−1^ and stored in liquid nitrogen.

### Crystallization of native intact complex I

Intact *T. thermophilus* complex I crystals were obtained using sitting drop crystallization at 23 °C. Purified complex I (diluted to 18–20 mg mL^−1^ in buffer A) was incubated with additional TDM, reaching a final concentration of 4% (w/v) TDM. Previously reported crystallization conditions^16^ were optimized by the addition of heterologously expressed and purified subunit Nqo16 to the complex. Otherwise, only a small proportion of the purified enzyme contains Nqo16 and the rest does not crystallize. Addition of Nqo16 allowed us to utilize the entire pool of the purified enzyme for crystallization, as needed for protein-consuming multiple soaking experiments. The ratio of added Nqo16 to complex I was optimized in test crystallizations to 1:0.2 (CXI:Nqo16) and then, such complex I/Nqo16 mixture, was mixed at a ratio of 2:1 (v/v) with a mini-screen of crystallization solutions, containing 100 mM Bis–Tris pH 6.0, a gradient of 19–25% (w/v) PEG 4000, 100 mM KCl, 100 mM glutaric acid, and up to 12 different detergents, all of them promoting the crystal growth to different extents. Rod-shaped crystals (50 × 50 × 500–700 μm) were obtained after 1–3 weeks, cryoprotected in 100 mM Bis–Tris pH 6.0, 9% PEG 4000, 50 mM KCl, 50 mM glutaric acid, 25% ethylene glycol, and 0.01% TDM before storing in liquid nitrogen.

### Crystallization in the presence of ligands

Complex I structure containing DQ was determined from an intact crystal obtained after 24 days in 100 mM Bis–Tris pH 6.0, 23% (w/v) PEG 4000, 100 mM KCl, 100 mM glutaric acid, and 50 mM 3-(1-pyridinio)-1-propanesulfonate (NDSB-201, Santa Cruz Biotechnology), soaked overnight in the cryoprotectant solution containing 500 μM DQ and 1% ethanol. In the case of piericidin A, aureothin, and pyridaben, a combination of co-crystallization and soaking was performed. Intact complex I was mixed with 50 μM piericidin A in 1% DMSO (1:1 ratio) obtaining the best co-crystals after 14 days in 100 mM Bis–Tris pH 6.0, 24% (w/v) PEG 4000, 100 mM KCl, 100 mM glutaric acid, and 0.59 mM *n*-undecyl-β-maltoside (UDM, Glycon). These co-crystals were further soaked in the cryoprotectant solution with 100 μM piericidin A and 1% DMSO for 4 h. Mixture of 100 μM aureothin in 1% DMSO with intact complex I (1:1 ratio) resulted in co-crystals grown for 8 days in 100 mM Bis–Tris pH 6.0, 25% (w/v) PEG 4000, 100 mM KCl, 100 mM glutaric acid, and 7.6 mM 4-cyclohexyl-1-butyl-β-d-maltoside (CYMAL-4, Anatrace), further soaked in cryoprotectant solution with 100 μM aureothin in 1% DMSO for 30 min. Intact complex I was mixed with 150 μM pyridaben in 1% DMSO (1:1 ratio), obtaining the best co-crystals after 15 days in 100 mM Bis–Tris pH 6.0, 21% (w/v) PEG 4000, 100 mM KCl, 100 mM glutaric acid, and 1.02 mM octyl-maltoside fluorinated (Anatrace), further soaked in 200 μM pyridaben and 1% DMSO for 6 min. Finally, to obtain complex I in the presence of NADH under reducing conditions, crystallization experiments were performed inside an anaerobic glove box (Belle Technology). Best intact complex I crystals, grown after 13 days in 100 mM Bis–Tris pH 6.0, 23% (w/v) PEG 4000, 100 mM KCl, 100 mM glutaric acid, and 0.01 *n*-tetradecyl-β-d-maltopyranoside (T315S, Anatrace), were soaked in cryoprotectant solution containing 20 mM NADH and 20 mM sodium dithionite for 4.5 min.

### X-ray data collection and processing

Diffraction data from crystals containing intact complex I in the presence of ligands was collected at 100 K in beamlines ID29 and ID23-2 at the European Synchrotron Radiation Facility (ESRF, Grenoble, France) and in beamline I03 at Diamond Light Source (DLS, Oxford, UK) (Supplementary Table 1). Diffraction images were processed in XDS and XSCALE^52^. After soaking in different ligands, all crystals showed a similar unit cell and belonged to the P2_1_ space group, containing 2 mol/ASU (about 69% of solvent content) and strongly pseudo-merohedrally twinned (twin fractions of 0.47–0.50). Resolution limits ranged from 3.1 to 3.6 Å, depending on the ligand and the dataset. However, weak diffraction along *b* axis was a common feature observed in all intact complex I crystals, requiring anisotropic scaling and truncation at *F/σ* = 2.5 along a*, b* and c* axes (Diffraction Anisotropy Server, UCLA). The starting structure constituted previously published structure of complex I^16^ with Nqo6 55-70 loop re-built according to the improved density from the 11 merged isomorphous native datasets (Supplementary Table 1). This model was used for molecular replacement in Phaser^53^. All structures were refined in *phenix.refine*^54^ following the same protocol, until *R*-factors convergence, to assess the reliability of differences observed among them. The ligands were modelled only in cases where strong Fo–Fc and 2Fo–Fc density (calculated before the placement of any ligand in the model) clearly indicated the presence of a bound ligand. Model building was performed in Coot^55^ and all structures have been validated in MolProbity^56^.

### Electron microscopy

Intact complex I from *T. thermophilus* was purified as described above. The more abundant, non-crystallisable, form of complex I was used for specimen preparation, as Nqo16 is not needed for redox or proton-pumping activity, and so we did not consider addition of Nqo16 essential for cryo-EM. After particle spread optimization, grids were prepared with complex I solubilized in a buffer composed of 20 mM Bis–Tris pH 6.0, 75 mM NaCl, 2 mM CaCl_2_, 0.01% [w/v] of DDM, 0.5% [v/v] of TDM, and 2.5–3.25% [v/v] of glycerol. With the aim of trapping complex I in its oxidized and reduced states, specimen grids were prepared with the addition of either 5 mM NAD^+^ or 5 mM NADH just before applying sample to the grids. The sample (~2.7 μL) was added to Quantifoil R0.6/1.0 300 mesh grids in Vitrobot MKIII (RH 100%, *T* = 22 °C). Subsequently specimens were blotted, flash-cooled in liquid ethane and stored in LN_2_ until usage.

### Image collection and processing for EM

Automated data collection was performed using a 300 kV FEI Krios TEM equipped with a Falcon-II DED in the Laboratory of Molecular Biology (LMB-MRC, Cambridge, UK). 710 micrographs of complex I in presence of NAD^+^ were acquired in one session. 2184 micrographs of complex I in presence of NADH were acquired in three different sessions. A single image per hole was acquired with magnification of ×81,395 corresponding to a 1.72 Å pixel size. Images consisted of 34 movie frames captured during an exposure period of 2 s with an electron dose-rate 17 e^−^ Å^−2^ s^−1^. The defocus ranged from 2.5 μm up to 4 μm.

All the image-processing steps were performed using RELION2.0^57^. Unless otherwise stated, the same strategy for data processing and settings were applied to both the NADH and the NAD^+^ datasets. The MOTIONCORR^58^ was applied to both series of micrographs to account for whole-frame drift. CTF parameters were calculated using Gctf with subsequent local refinement for each particle^59^. Then, a subset of manually picked particles was used to run a preliminary 2D-classification for the selection of the reference 2D classes, which were used as templates in the automated particles picking. Particle extraction was carried out with a box size of 256 pixel. A total of 149k and 336k particles were picked from the NAD^+^ and NADH datasets, respectively, and classified into 100 (NAD^+^ subset) or 150 (NADH subset) 2D classes. Particles from the best 2D classes were selected and subjected to 3D classification with regularization parameter *T* = 8. The initial 3D reference map was prepared by low pass-filtering (30 Å) the intact complex I model from *T. thermophilus* (PDB 4HEA [10.2210/pdb4HEA/pdb])^16^ without Nqo16.

For the NADH dataset, particles were divided initially into four 3D classes. Particles from the good class (class 4) were re-classified into three classes, and then two good classes were taken forward. The most populated class (class 2), named NADH major, contained 44,511 particles; the second most populated class (class 1), named NADH minor, contained 27,908 particles.

For the NAD^+^ dataset, the full particle ensemble was classified into four 3D classes and, in parallel, into six 3D classes. The first 3D classification resulted in two well-defined classes, class 2 (NAD^+^ major) and class 3 (NAD^+^ minor) of 38,659 and 20,840 particles, respectively. The second type of 3D classification resulted also in two well-defined classes (classes 1 and 2). Particles not included in the classes mentioned above were discarded. Selected particles, classes 1 and 2, were combined in one ensemble and processed for a second round of 3D classification into three classes. At this stage, the major class (class 2) comprised 40,831 particles and the minor class (class 1) was of 24,185 particles. After comparing the particle content of the major and minor classes obtained so far, it was decided to pool all the particles from classes 2 and 3 of the first run with particles from classes 1 and 2 from the second 3D classification cascade. The resulting ensemble contained 77,535 unique particles after removing duplicates, which were re-classified into two 3D classes. Class 1, named as NAD^+^ major, contained 47,850 and class 2 (NAD^+^ minor) contained 29,685.

The processing of both NAD^+^ and NADH datasets yielded a total of four classes: one major class and one minor class for complex I with NAD^+^ and one major and one minor with NADH. For automated 3D refinement particles from each subset were re-extracted with a 512 pixel box and refinement was performed separately for each class, followed by particle polishing^60^. Finally, polished particles were refined and the EM density map for each class was sharpened with either automatically estimated or manually set *B*-factor.

### Cryo-EM model building and refinement

Four electron density maps were used to build structural models of four different conformational states of complex I. Maps for minor states with bound NAD^+^ and NADH, that is, CXI_MN:NAD+_ and CXI_MN:NADH_, respectively, are characterized by resolution of 5.5 and 6.1 Å, compared to the 4.3 and 4.25 Å resolution maps, which were obtained for major states with NAD^+^ and NADH, respectively. The structures of minor states were built using the molecular dynamics flexible fitting (MDFF) method^61^, which is more suitable for maps of medium resolution (implemented in NAMD code^62^). For the structures of major states, initially both the MDFF approach and the density-guided optimization protocol implemented in Rosetta code^63^ were pursued. The details of the applied Rosetta protocol are described in our previous study^17^. In brief, we produced 100 models for each subunit of complex, using the *elec_dens_fast* function (with –*denswt* = 40), which was then followed by structure relaxation, using the –*FastRelax* flag, selection of the best fitting structure, and manual inspection of the best model in COOT. The final models for the 15 subunits of the complex I were then assembled into one model and its structure was globally optimized, using the protocol which allowed only for minor movements of side-chains (backbone restrained), resulting in the removal of inter-atomic clashes and improved fit of side-chains. The second of the applied protocols, the MDFF, incorporates an external potential (added as an additional term to the potential energy function of MD simulation), which is derived from the map and introduced into molecular dynamics simulation to steer atoms into high-density regions. The MDFF was performed with the CHARMM 22^64,65^ force field at 300 K, with time step of 2 fs, with the scaling factor for the applied external potential of 0.3 kcal/mol for molecular dynamics part (chosen from several trials), and with the scaling factor of 5 kcal/mol (chosen from several trials) for energy minimization. The stereochemical quality of the fitted structures was preserved by restraining their heavy atoms with a small force constant of 1 kcal/mol and by restraining ϕ and ψ dihedral angles of amino-acid residues in helices and β strands, which effectively preserved the secondary structure of complex, at the same time leaving model fully flexible. The quality of fit for Rosetta-derived and MDFF-derived models was assessed, using the Time-line analysis plugin (implemented in VMD^66^), by calculating the cross-correlation coefficient (cc) between simulated map and the experimental map. Models of major states which were produced by MDFF were characterized by better density fit and by lower clash score (MolProbity) than the ones obtained in Rosetta. The obtained density fit scores show that the regions of a worse fit correspond mainly to flexible peripheral parts of Nqo12 subunit and peripheral regions of PA. Therefore, the MDFF models were chosen as final models of major states used for analysis.

### Structural and bioinformatic analyses

All the structural analyses in this work were performed with the usage of PyMOL, VMD, and PROSMART codes^67^. The interior of the studied structures was solvated with the usage of Dowser code^49^ with default parameters and protonation states of amino acid residues were assigned in PROPKA code^48^. NMA was performed using Bio3D v2.3 package^68^, using two elastic network models (ENM), the ANM (cut-off 15 Å)^69^ and HCA^70^. Network analysis of allosteric couplings between respective regions of complex I was performed based on generated NMA trajectories. We constructed several networks based on the trajectories obtained from NMA, using the edge betweenness algorithm, with a correlation cut-off of 0.40–0.70 (with increment of 0.05) for HCA force field, and with correlation cut-off 0.10–0.70 (with increment of 0.10) for ANM force field. The HCA-based networks constructed with cut-off 0.40–0.50 had similar structures to the ones obtained with ANM force field and cut-off of 0.20. For clarity, in this work we refer to HCA-based networks, which were obtained with cut-off 0.45.

The coarse-grained (CG) molecular dynamics simulations were performed with Martini v.2.2 model^45^. The structure of the protein was additionally restrained with the Elastic Network model (ElNeDyn) version 22^71^, which was shown to effectively maintain the conformation of a protein while keeping its internal dynamics. The quinone parameterization is according to the earlier studies^72^ and represents a standard combination of P1 and Na beads in all configurations with the exception of the UQ2^−^ configuration, where P1 beads were replaced by Qda beads to simulate the ubiquinol dianion form. In the UQ, UQ2^−^, or UQH2 configurations SC3 beads of His38 and Tyr87 were given SQd types without negative charges. For direct comparisons, in the UQH2_His38^−^_Tyr87^−^ configuration the SC3 beads of His38 and Tyr87 were both SQd bead type and given −1.0 charge. The Asp139 was assigned in all configurations to be composed of Na/Qa beads (charge −1.0), i.e. default parameters. The structure of protein was inserted into a DOPC bilayer patch of a size of 340 (*x*) and 150 (*y*) angstroms and then shortly equilibrated for 10 ps. The equilibration phase was followed by the production simulations, which all lasted 2 µs and were performed with 20 fs time step. We performed two independent simulations for each simulated system, starting with a different random seed and from different initial temperature. For the network analysis in Fig. 8 and Supplementary Fig. 9, 2000 snapshots collected every 1 ns were used to make network models.

### Reporting summary

Further information on experimental design is available in the Nature Research Reporting Summary linked to this paper.

## Supplementary information

Supplementary Information

Description of Additional Supplementary Files

Supplementary Data 1

Reporting Summary

## Data Availability

X-ray data and models are deposited in the PDB under accession codes 6Y11 (INT), 6I1P (NADH), 6I0D (DQ), 6Q8O (PIE), 6Q8W (AUT), and 6Q8X (PYR). Cryo-EM maps and models are deposited in EMDB/PDB under accession codes: NADH dataset, major state EMD-11231, PDB ID 6ZIY; NADH dataset, minor state EMD-11237, PDB ID 6ZJN; NAD^+^ dataset, major state EMD-11235, PDB ID 6ZJL; and NAD^+^ dataset, minor state EMD-11238, PDB ID 6ZJY. Other data are available from the corresponding author upon reasonable request.
