# Supplementary material for: Key role of quinone in the mechanism of respiratory complex I
Source: Nat Commun. 2020 Aug 18;11:4135. doi: 10.1038/s41467-020-17957-0 (PMC7434922; doi:10.1038/s41467-020-17957-0)
Supplement: Supplementary file 1 — Supplementary Information [file 41467_2020_17957_MOESM1_ESM.pdf]

Supplementary Information

Key role of quinone in the mechanism of respiratory complex I

J. Gutiérrez-Fernández, K. Kaszuba et al.

3 Supplementary Tables

9 Supplementary Figures

Supplementary Table 1. X-ray data collection and model refinement statistics

Structure	#CXI_{INT}	CXI:NADH	CXI:DQ	CXI:PIE	CXI:AUT	CXI:PYR
PDB ID	6Y11	6I1P	6I0D	6Q8O	6Q8W	6Q8X
Data collection						
Beamline	Merged data	I03	ID29	ID29	ID23-2	I03
Space group	$P2_1$	$P2_1$	$P2_1$	$P2_1$	$P2_1$	$P2_1$
Cell dimensions						
a, *b*, *c* (Å)	96.3, 340.9, 263.3	96.1, 341.5, 263.9	94.7, 328.0, 260.9	95.1, 336.6, 262.8	96.0, 340.5, 264.3	95.2, 338.6, 263.2
α, β, γ (º)	90, 100.57, 90	90, 100.52, 90	90, 100.40, 90	90, 100.41, 90	90, 100.44, 90	90, 100.41, 90
Twin fraction	0.49	0.47	0.49	0.49	0.49	0.49
Wavelength (Å)	0.9000	0.9000	0.9000	0.9000	0.9000	0.9000
Scaling						
Resolution (Å)	58-3.3 (3.36-3.30)	59-3.5 (3.69-3.50)	49-4.0 (4.22-4.00)	40-3.8 (4.01-3.80)	50-3.5 (3.69-3.50)	58-3.8 (4.01-3.80)
Resolution at $CC_{1/2}$ = 0.3 (Å)	3.10	3.31	3.96	3.60	3.42	3.73
R_{sym} or R_{merge} (%)	26.7 (429.3)	12.7 (90.8)	19.1 (70.7)	13.0 (77.0)	22.8 (129.8)	12.5 (94.8)
R_{pim} (%)	5.8 (96.6)	9.0 (66.2)	17.5 (65.4)	11.1 (68.3)	7.8 (63.2)	9.7 (74.1)
CC_Imean	0.998 (0.552)	0.996 (0.592)	0.949 (0.277)	0.995 (0.633)	0.992 (0.385)	0.997 (0.324)
Mn(I/σI)	7.9 (1.1)	6.7 (1.4)	5.3 (1.5)	5.2 (1.1)	11.4 (1.5)	6.2 (1.3)
Completeness (%)	100 (100)	96.9 (93.8)	96.3 (97.0)	95.9 (96.6)	96.2 (88.2)	96.6 (94.7)
Multiplicity	22.2 (20.0)	2.7 (2.5)	2.0 (2.0)	2.1 (2.1)	8.7 (4.6)	2.3 (2.2)
Anisotropic truncation limits, Å, along *a**, *b** and *c**	3.1, 3.4, 3.1	3.4, 3.8, 3.2	3.6, 4.5, 3.6	3.6, 4.2, 3.7	3.5, 3.9, 3.4	3.5, 4.0, 3.6
Completeness after aniso truncation (%)	100 (23)	76.1 (6.1)	76.4 (20.7)	79.9 (16.8)	84.1 (19.5)	81.7 (13.3)
Refinement						
Resolution (Å)	59-3.1	59-3.2	30-3.6	49-3.6	50-3.4	58-3.5
No. reflections	268255	207039	137819	148790	191608	166363
R_{work}/ R_{free} (%)	20.4 / 22.9	21.0 / 23.1	21.4 / 22.5	21.8 / 23.5	21.3 / 23.4	21.5 / 23.2
No. atoms	74086	74174	74132	74146	74144	74134
Protein	73896	73896	73896	73896	73896	73896
Ligand	190	278	236	250	248	238
B factors ($Å^{-2}$)						
Protein	101.0	74.2	80.4	111.9	63.9	97.4
Ligand	97.9	54.3	75.5	85.7	45.4	76.6

*Highest resolution shell is shown in parenthesis.

R_{pim}, *CC_Imean* (cross-correlation between two random half-data-sets in SCALA[73]) and anisotropic F/σ were used as main criteria for resolution estimation, due to high multiplicity and/or anisotropy of data.

#Diffraction data for CXI_{INT} structure is from 11 merged isomorphous native datasets.

Supplementary Table 2. Cryo-EM data collection and model refinement statistics

Structure	MJ:NADH	MN:NADH	MJ:NAD$^+$	MN:NAD$^+$
PDB ID	6ZIY	6ZJN	6ZJL	6ZJY
EMDB ID	EMD-11231	EMD-11237	EMD-11235	EMD-11238
Data collection and processing				
Magnification	81,395x	81,395x	81,395x	81,395x
Voltage (kV)	300	300	300	300
Electron exposure (e–/Å^2)	34	34	34	34
Defocus range (μm)	-2.5 to -4.0	-2.5 to -4.0	-2.5 to -4.0	-2.5 to -4.0
Pixel size (Å)	1.72	1.72	1.72	1.72
Symmetry imposed	C1	C1	C1	C1
Initial particle images (no.)	205K	205K	91K	91K
Final particle images (no.)	44.5K	28K	48K	29K
Map resolution (Å)	4.25	6.11	4.29	5.53
FSC threshold 0.143				
Map resolution range (Å)	4-5	6-7	4-5	5-7
Refinement				
Initial model used (PDB code)	6Y11	6Y11	6Y11	6Y11
Model fitting into cryo-EM maps was performed using MDFF implemented in NAMD				
Model resolution (Å)	4.25	6.11	4.29	5.53
Map sharpening *B* factor (Å^2)	-100	-105	-100	-111
Model composition				
Non-hydrogen atoms	36129	Model	36085	Model
Protein residues	4648	trimmed to C_β	4648	trimmed to C_β
Ligands	9 FeS, FMN, NADH		9 FeS, FMN	
B factors (Å^2)				
Protein	N/A		N/A	
Ligand				
R.m.s. deviations				
Bond lengths (Å)	0.021		0.022	
Bond angles (°)	2.41		2.38	
Validation				
MolProbity score	2.03		1.70	
Clashscore	2.37		1.01	
Poor rotamers (%)	3.25		2.43	
Ramachandran plot				
Favored (%)	86.75		87.51	
Allowed (%	9.76		9.57	
Disallowed (%)	3.49		2.92	

Supplementary Table 3. IC_{50} values obtained for inhibitors of *T. thermophilus* complex I in NADH:DQ activity assays.

Inhibitor	IC_{50}
Piericidin A	31 nM
Rotenone	190 nM
Aureothin	10.3 nM
Rolliniastatin-1	930 nM
Pyridaben	15.6 nM
Fenpyroximate	630 nM
Myxothiazol	2.5 µM
Stigmatellin	60 µM
HQNO	7.4 µM
Capsaicin	197 µM

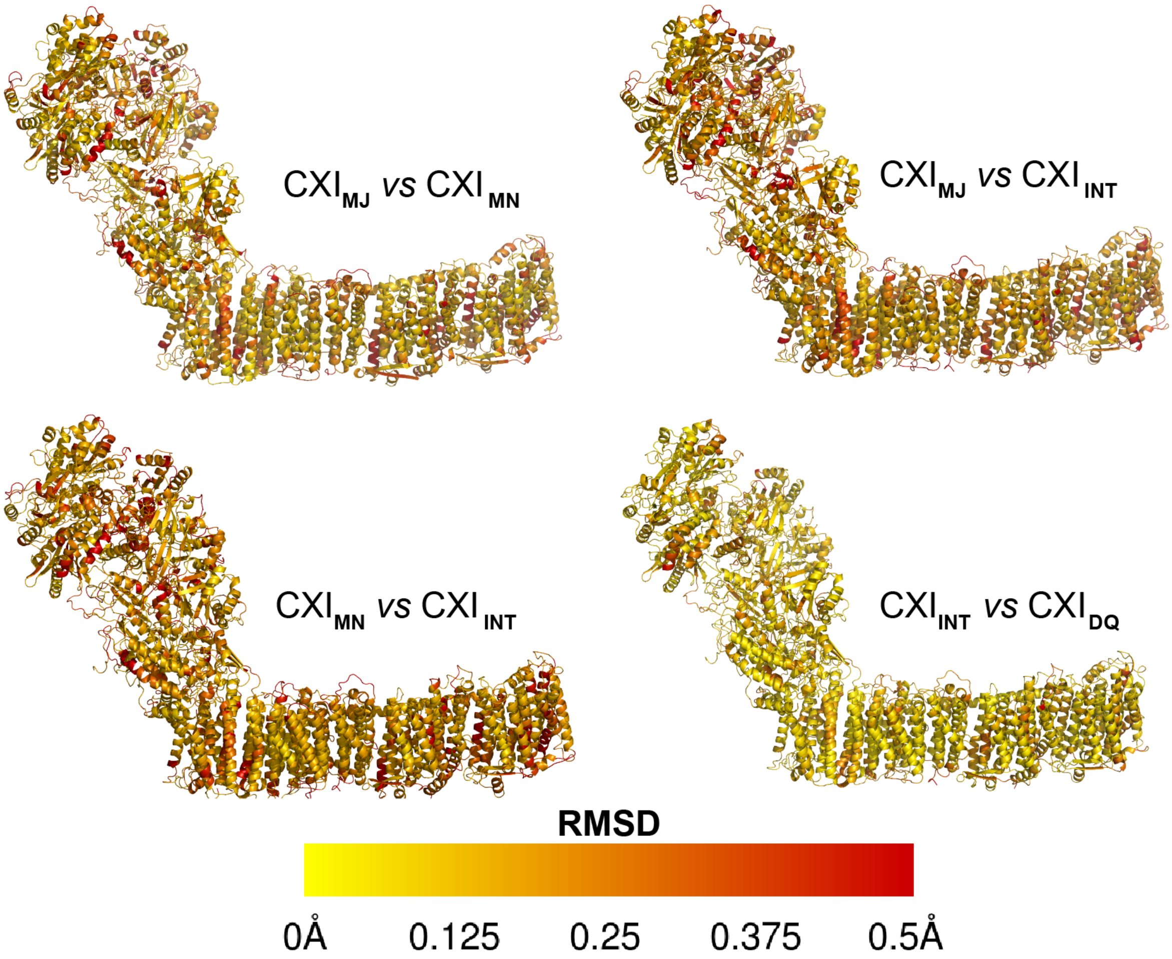

Supplementary Fig. 1. The analysis of local structural conservation of all compared configurations.

Compared structures are coloured by local conservation of a backbone, expressed in RMSD, which is computed for the main-chain atoms, the so-called "flexible score". All compared structures display a characteristic lack of conservation, accumulating mainly in the regions of PA and in the first proton pump, the E-channel. The analysis of local conservation was performed with the usage of PROSMART code with window size 7[67].

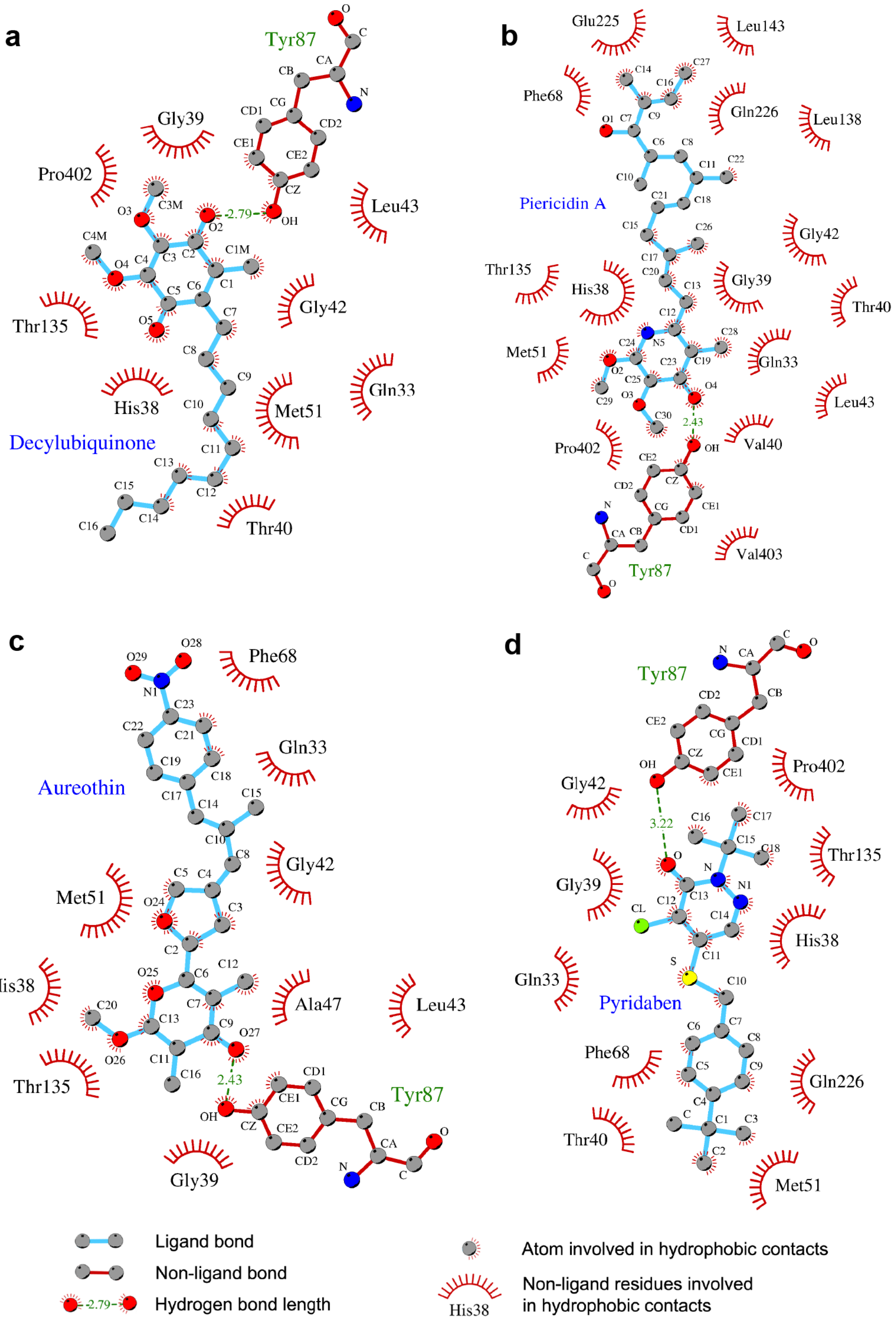

Supplementary Fig. 2. Quinone-like ligands interactions at the Q-site.
Schematic diagram, performed with LIGPLOT[74], showing the surrounding environment of DQ (**a**), piericidin A (**b**), aureothin (**c**) and pyridaben (**d**) bound to CXI in the Q-site. With the exception of $Y87_4$ that establishes a hydrogen bond interaction with the quinone moiety of every ligand, all the remaining residues are stabilized through hydrophobic contacts.

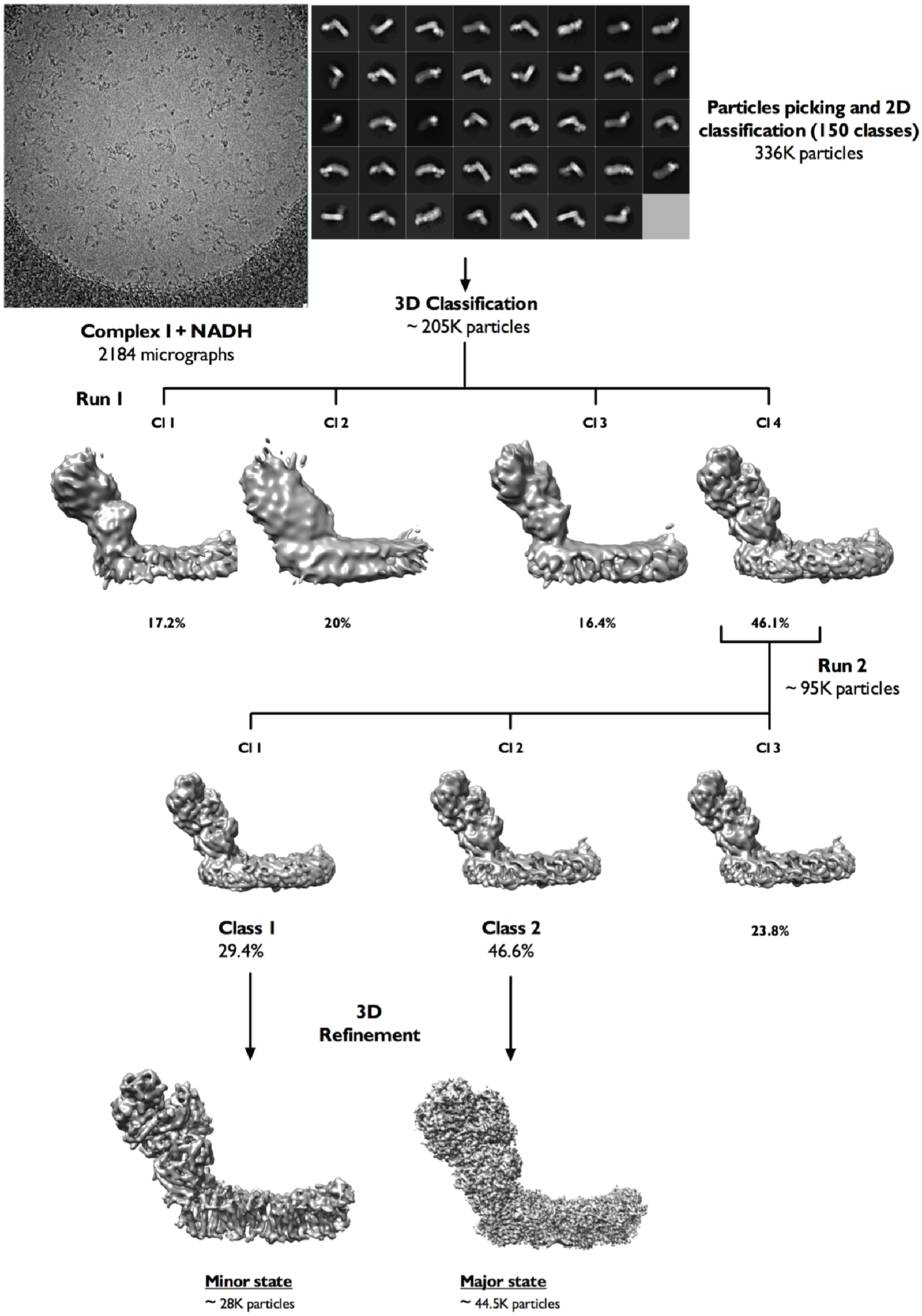

Supplementary Fig. 3. Cryo-EM processing strategy, NADH dataset.
A typical micrograph is shown at the top left side. At the top right side, the best 2D classes after 2D classification of the auto-picked particles are shown. The selected particles underwent an iterative procedure of 3D classification and refinement as shown in the chart

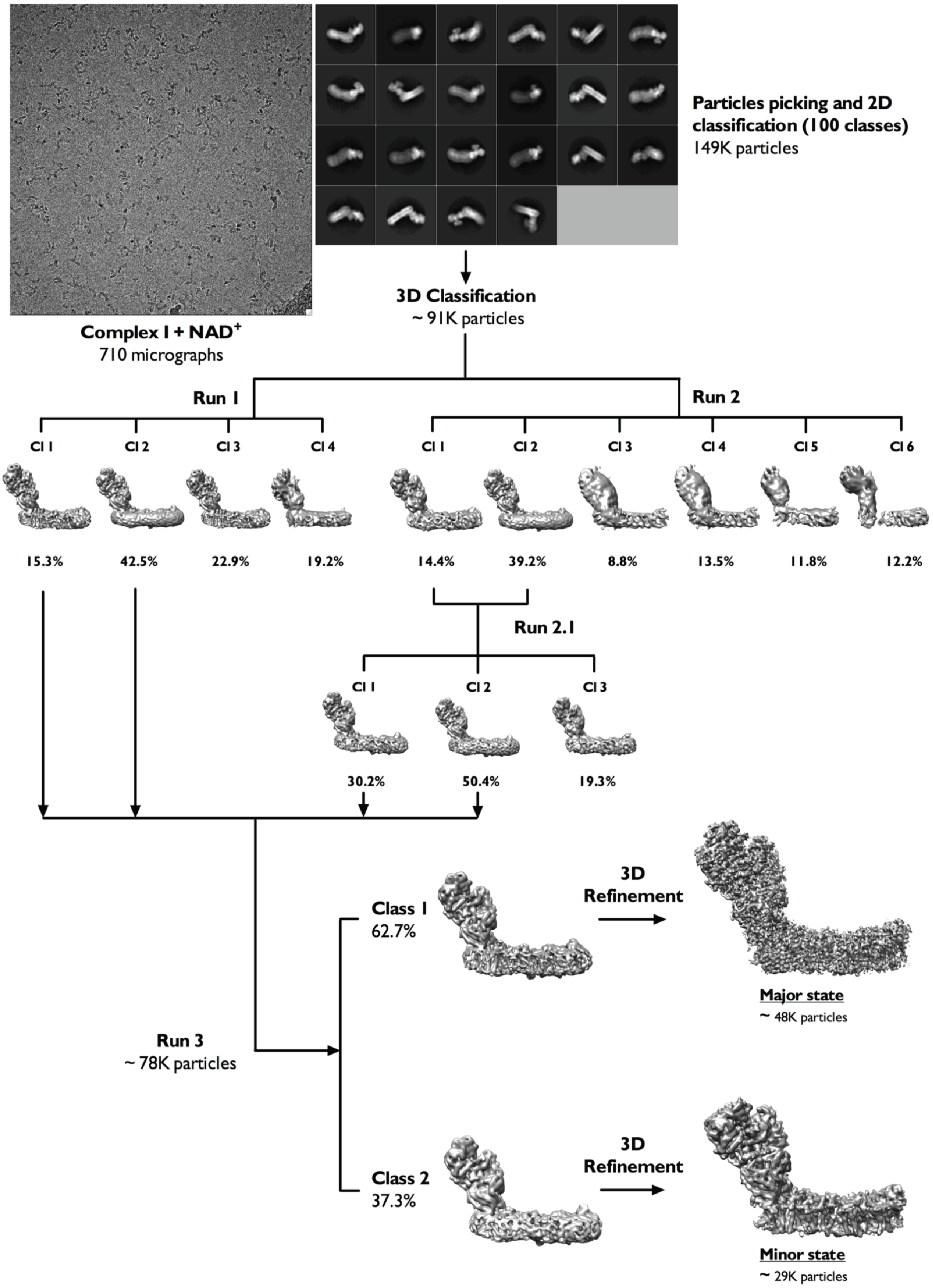

Supplementary Fig. 4. Cryo-EM processing strategy, NAD$^+$ dataset.
A typical micrograph is shown at the top left side. At the top right side, the best 2D classes after 2D classification of the auto-picked particles are shown. The selected particles underwent an iterative procedure of 3D classification and refinement as shown in the chart.

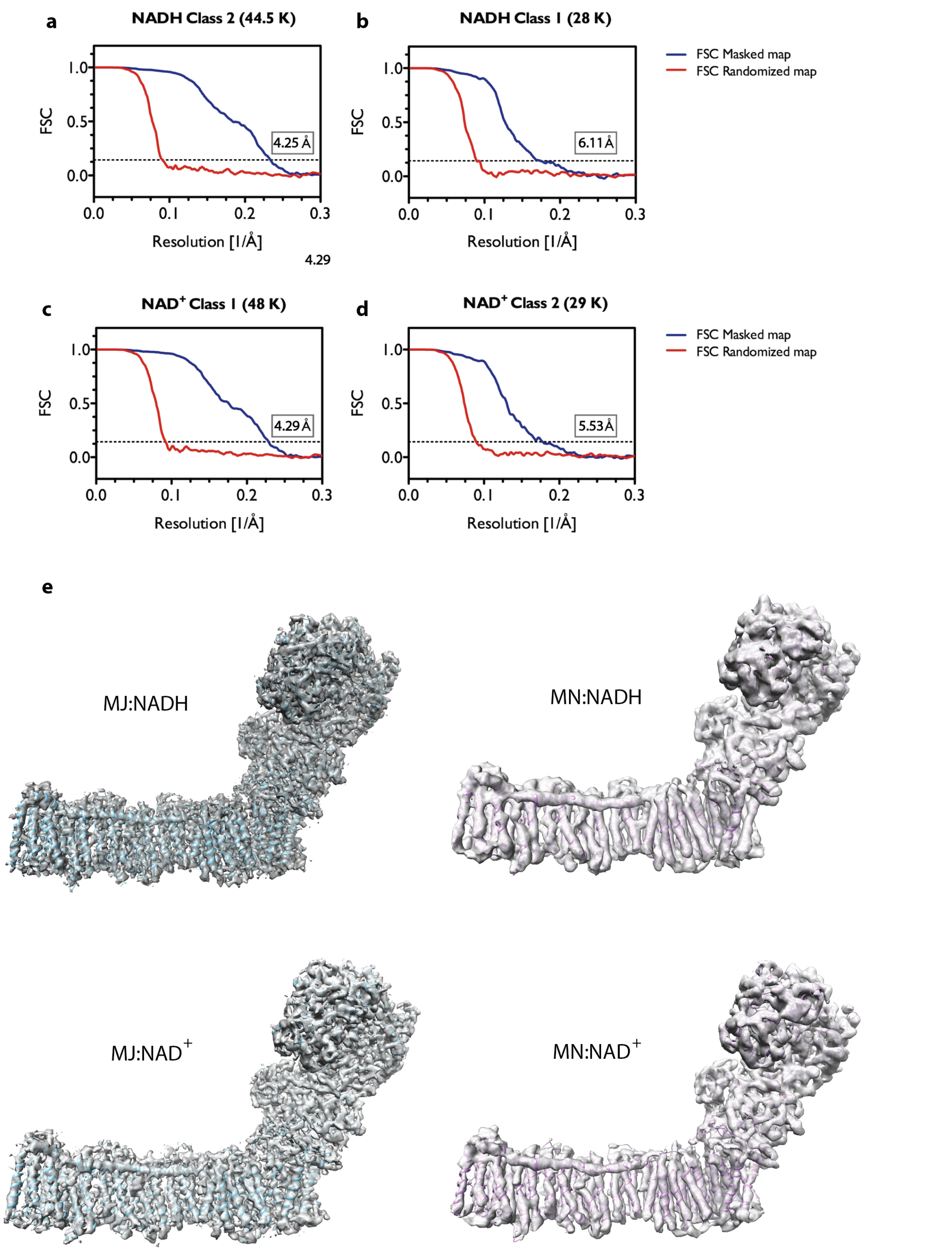

Supplementary Fig. 5. Gold-standard Fourier Shell Correlation (FSC) cryo-EM refinement curves. Dashed lines indicate the threshold at 0.143. The blue and red curves represent the FSC for the masked and randomized maps respectively. **a,** Class 2 (major) of the NADH dataset. **b,** Class 1 (minor) of the NADH dataset. **c,** Class 1 (major) of the NAD^+ dataset. **d,** Class 2 (minor) of the NAD^+ dataset. **e,** Cryo-EM density for each class, with fitted models shown as cartoon.

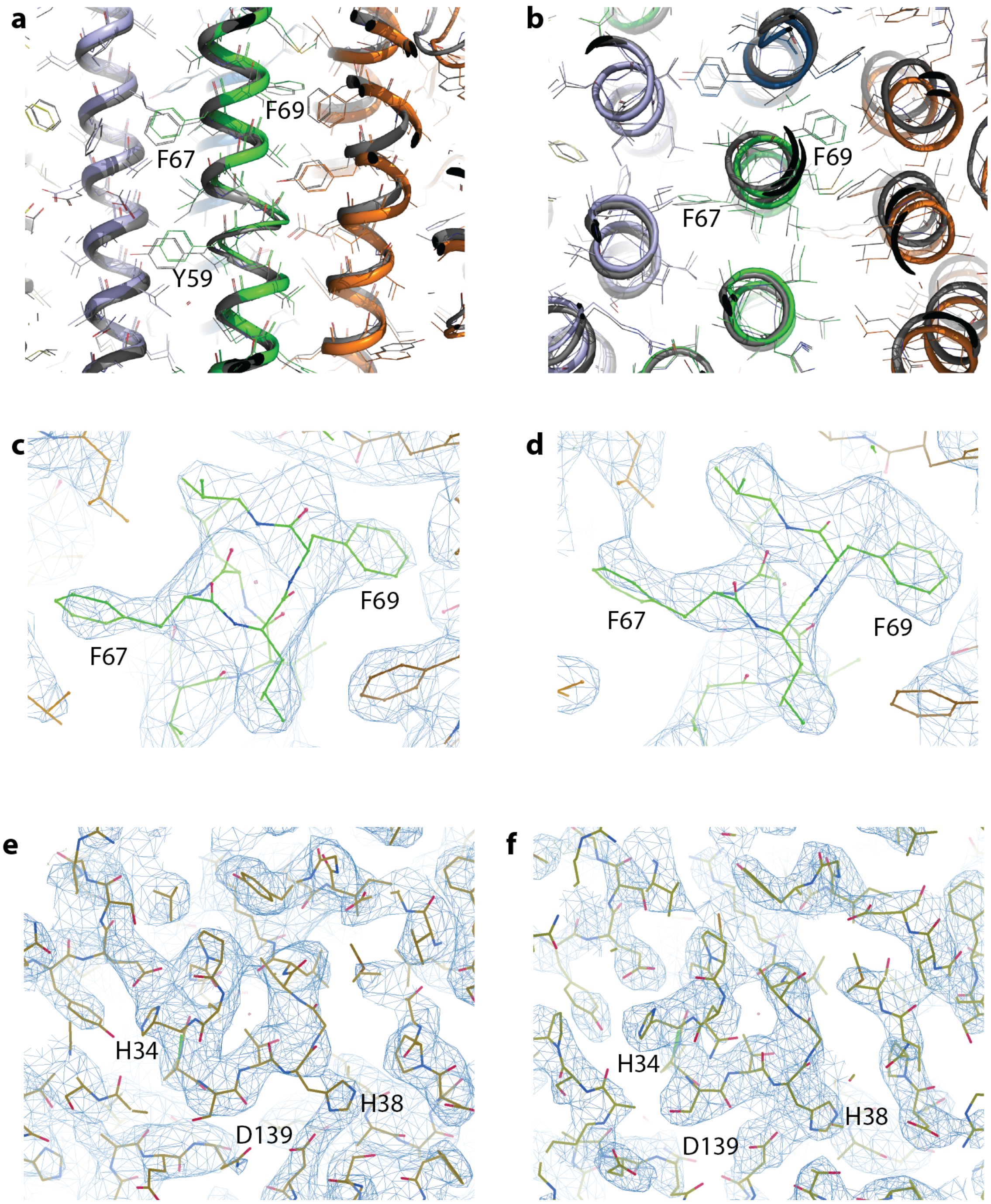

Supplementary Fig. 6. a-d, Conformation of key Nqo10 helix TM3 in native and DQ – bound structures. **a, b,** CXI_{INT} (gray) and CXI_{DQ} (coloured by subunit) structures are aligned by subunit Nqo10 (green), showing that conformational changes involve not only global but also local re-arrangements. Prominent Nqo10 residues are labelled. Note that in CXI_{DQ} structure TM3 changes its conformation from native starting from around π-bulge area (Y59) going up towards the C-terminus, resulting in a twist of about 30° and a tilt. **a** – side view, **b** – view from the cytoplasm. **c, d,** 2Fo-Fc maps and structures of the Nqo10 TM3 area where the conformation changes, for CXI_{INT} (**c**) and CXI_{DQ} (**d**). **e, f** 2Fo-Fc maps and structures of the Nqo4 β1-β2 loop with key Nqo4 residues labelled, for CXI_{INT} (**e**) and CXI_{DQ} (**f**). Maps in **c-f** demonstrate that structures can be refined reliably at current resolutions.

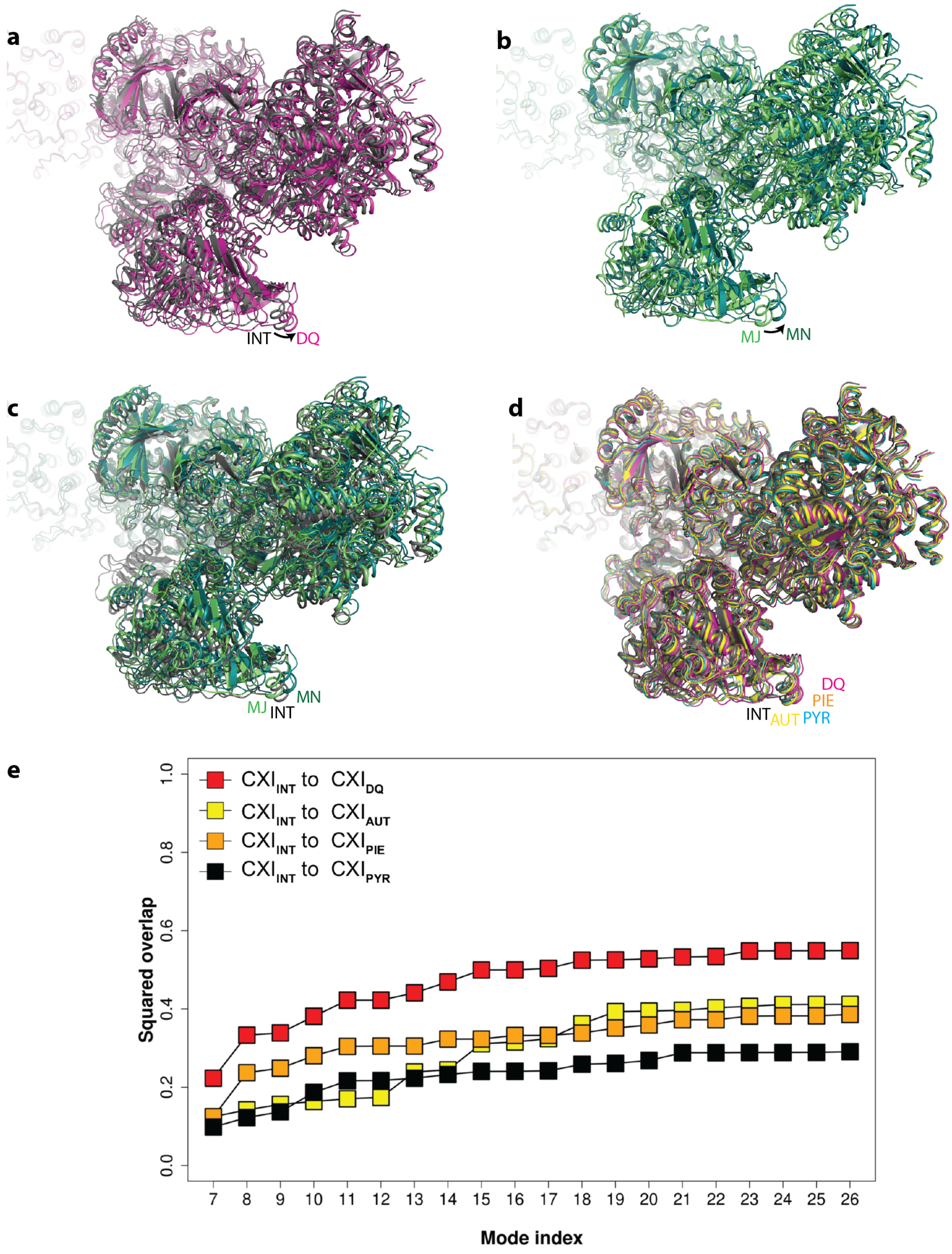

Supplementary Fig. 7. Comparison of structures. All structures were aligned to the membrane domain of CXI_{INT} structure and the view on the PA from the cytoplasm is shown, with MD to the left. The direction of the transition from one state to another is indicated by an arrow. **a**, CXI_{INT} (grey) and CXI_{DQ} (magenta) structures. **b**, $CXI_{MJ:NADH}$ (light green) and $CXI_{MN:NADH}$ (dark green) structures. Note that the transition from MJ to MN state is similar to that from INT to DQ (a). **c**, CXI_{INT}, $CXI_{MJ:NADH}$ and $CXI_{MN:NADH}$ structures. INT is more similar to MJ than to MN. **d,** Structures with quinone-like inhibitors bound show a range of transitions between INT (grey) and DQ (magenta) structures. CXI_{AUT} is in yellow, CXI_{PYR} in cyan and CXI_{PIE} in salmon. **e**, The overlap analysis (as in Fig. 6c) shows that transitions from CXI_{INT} structure to CXI_{AUT} and CXI_{PYR} involve relatively minor PA rotation component (mode 8), which is much stronger for CXI_{PIE} structure and is similar but accompanied in addition by a bending motion (mode 7) for CXI_{DQ} structure.

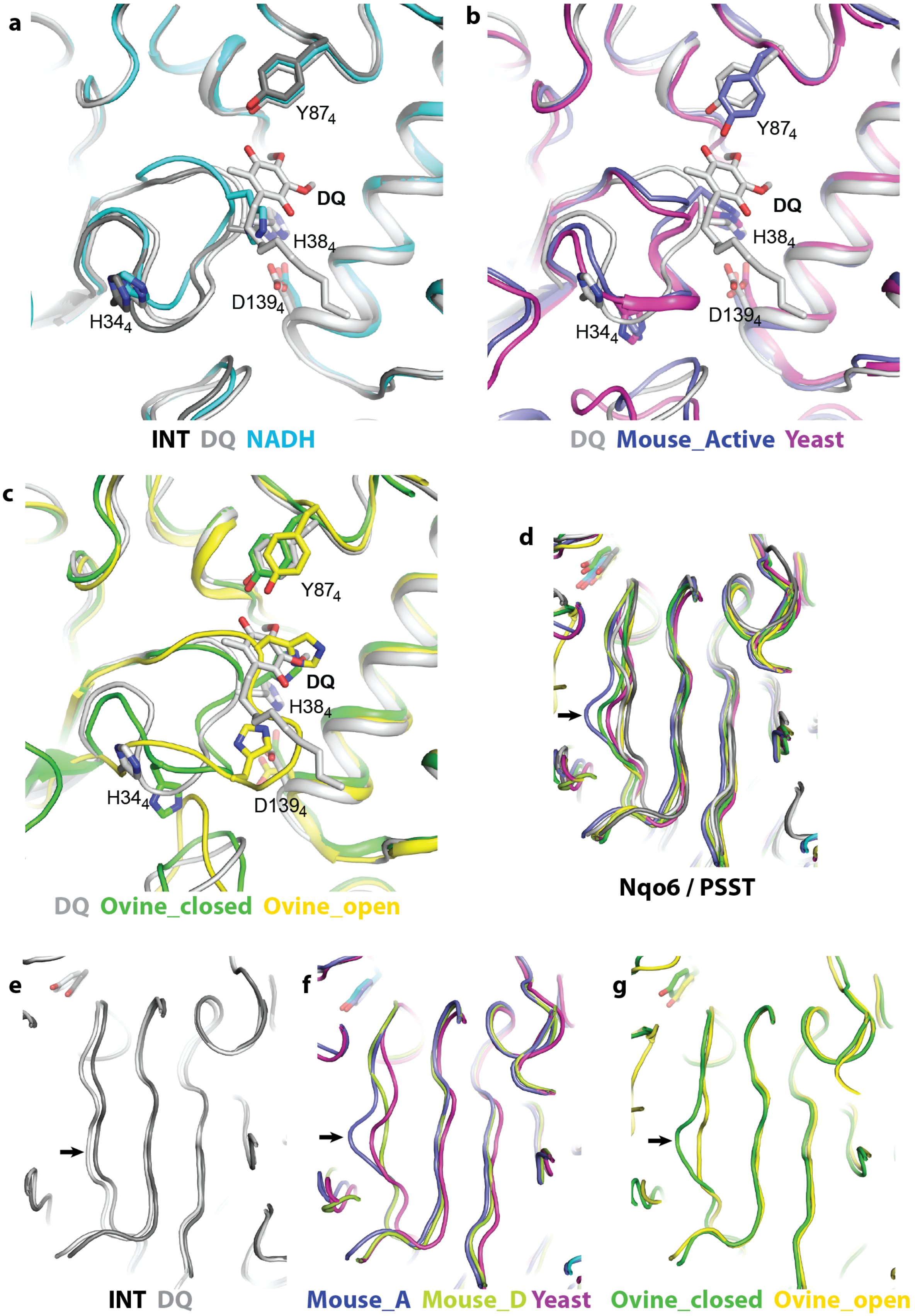

Supplementary Fig. 8. Comparison of the conformations of the key loops around Q cavity between the species. The legend is continued on the following page.

All structures were aligned by Nqo4 subunit. **a, b, c** Nqo4 β1-β2 loop. **a**, *T. thermophilus* CXI_{INT} (dark grey), CXI_{DQ} (light grey) and a structure after overnight soaking with NADH (cyan). Note that $H38_4$ from the NADH structure clashes with DQ position from the CXI_{DQ} structure (grey sticks). **b**, CXI_{DQ} (light grey), complex I from mouse in active state (PDB 6G2J, dark blue) and complex I from *Yarrowia lipolytica* (PDB 6RFR, magenta). Note roughly similar conformations of the loop, except for the orientation of $H34_4$ homologues in mitochondrial enzymes. **c**, CXI_{DQ} (light grey), ovine complex I in closed state (PDB 6QBX, green) and in open state (PDB 5LNK, yellow). Note that while the closed state is similar to other species in **b**, in open state the loop will clash with DQ position in *Thermus*. **d, e, f, g** The β-sheet of Nqo6 / PSST subunit. Different structures are coloured as above. Note that the first strand, flanking the Q cavity, undergoes the most conformational changes (**d**). They are concentrated around the conserved T40 from Nqo4, indicated by an arrow in **defg**. This area bulges out into the cavity in ovine closed and mouse active state enzyme as compared to ovine open and mouse deactive state (**f, g**). Yeast conformation is roughly between the two extremes (**f**), while in *T. thermophilus* the change is mostly the shift rather than re-arrangement (**e**).

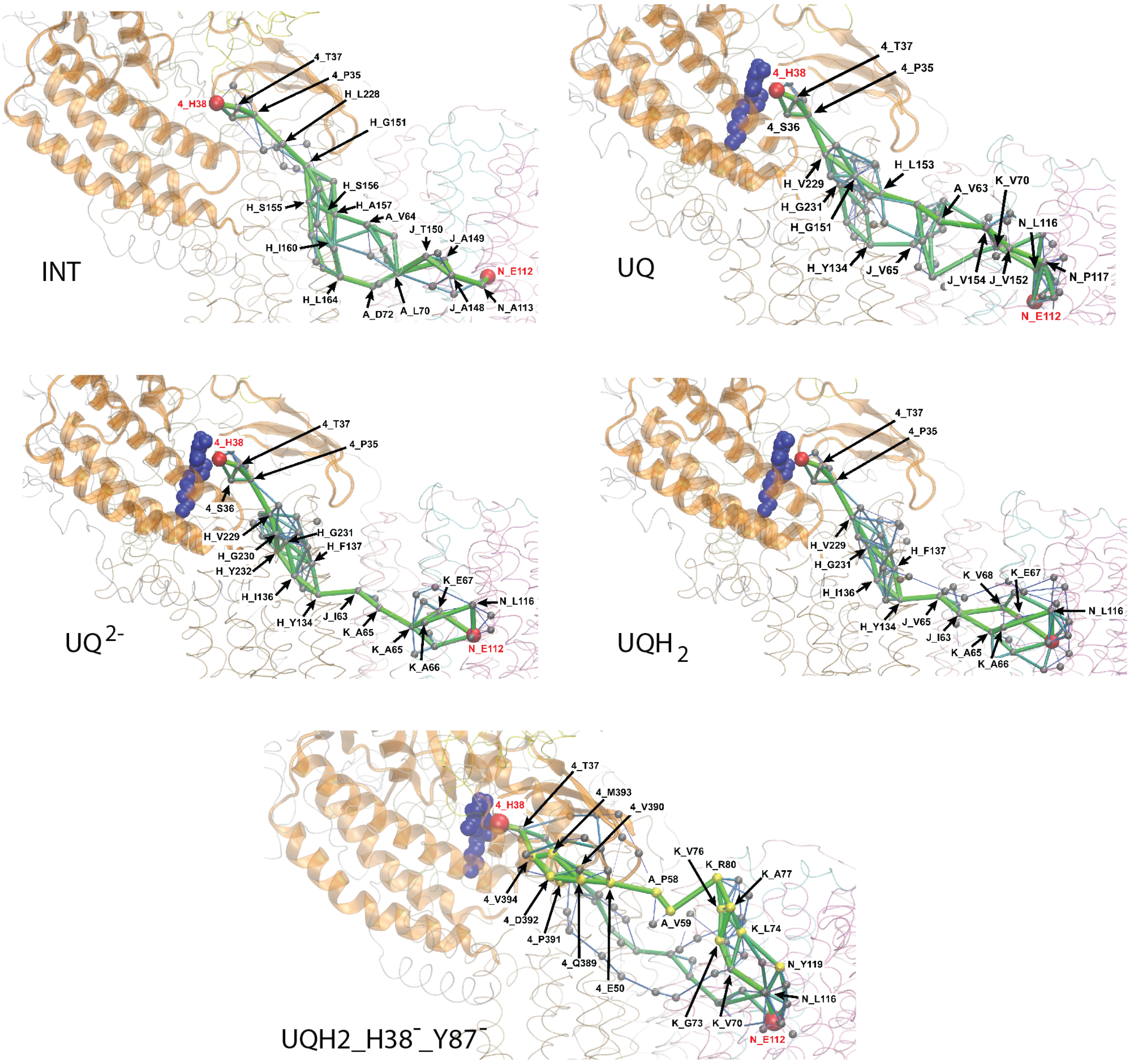

Supplementary Fig. 9. The network path analysis reveals the possible paths of allosteric signal propagation between the Q-site and the E-channel. The paths have been calculated between H38$_4$, coordinating quinone, and E112$_{14}$, a key residue in the proton channel of the nearest antiporter-like subunit Nqo14. The modelled coarse-grained systems included, as indicated, INT (no substrates), CXI$_{UQ}$, CXI$_{UQ2}^{-}$, CXI$_{UQH2}$ and CXI$_{UQH2_H38^-_Y87^-}$ states. The shortest, that is the most optimal paths (with the widths of linking edges proportional to the coupling strength) are in green, while the blue paths correspond to the less frequently "taken" suboptimal paths. Only the CXI$_{UQH2_H38^-_Y87^-}$ configuration features the unique path (with residues highlighted in yellow) between the Nqo4 β-sheet, neighbouring helix and E-channel. This unique path can be attributed to the H38$^-$/Y87$^-$ charge effect, while other systems feature paths that are closer to the one present in the INT system. Only residues with node degeneracy values of 0.3 and higher are labelled. Membrane domain residues are labelled in prefixes according to chain names in the structure, which correspond to Nqo8 as chain H, Nqo10 as J, Nqo7 as A, Nqo11 as K and Nqo14 as N.
