# Supplementary material for: Key role of quinone in the mechanism of respiratory complex I
Source: Nat Commun. 2020 Aug 18;11:4135. doi: 10.1038/s41467-020-17957-0 (PMC7434922; doi:10.1038/s41467-020-17957-0)
Supplement: Supplementary file 2 — Description of Additional Supplementary Files [file 41467_2020_17957_MOESM2_ESM.pdf]

Description of Additional Supplementary Files

File Name: Supplementary Data 1

Description: Network path analysis results. The node degeneracy values for each residue along the possible paths between $H38_4$ and $E112_{14}$ for the five systems displayed in Supplementary Fig. 9 are included. Highlighted in yellow are the residues from the unique path emerging in the charged system $CXI_{UQH2\_H38-\_Y87-}$.
